# Insights into the *Dekkera bruxellensis* Genomic Landscape: Comparative Genomics Reveals Variations in Ploidy and Nutrient Utilisation Potential amongst Wine Isolates

**DOI:** 10.1371/journal.pgen.1004161

**Published:** 2014-02-13

**Authors:** Anthony R. Borneman, Ryan Zeppel, Paul J. Chambers, Chris D. Curtin

**Affiliations:** 1 The Australian Wine Research Institute, Adelaide, Australia; 2 University of Adelaide, Adelaide, Australia; Harvard University, United States of America

## Abstract

The yeast *Dekkera bruxellensis* is a major contaminant of industrial fermentations, such as those used for the production of biofuel and wine, where it outlasts and, under some conditions, outcompetes the major industrial yeast *Saccharomyces cerevisiae*. In order to investigate the level of inter-strain variation that is present within this economically important species, the genomes of four diverse *D. bruxellensis* isolates were compared. While each of the four strains was shown to contain a core diploid genome, which is clearly sufficient for survival, two of the four isolates have a third haploid complement of chromosomes. The sequences of these additional haploid genomes were both highly divergent from those comprising the diploid core and divergent between the two triploid strains. Similar to examples in the *Saccharomyces* spp. clade, where some allotriploids have arisen on the basis of enhanced ability to survive a range of environmental conditions, it is likely these strains are products of two independent hybridisation events that may have involved multiple species or distinct sub-species of *Dekkera*. Interestingly these triploid strains represent the vast majority (92%) of isolates from across the Australian wine industry, suggesting that the additional set of chromosomes may confer a selective advantage in winery environments that has resulted in these hybrid strains all-but replacing their diploid counterparts in Australian winery settings. In addition to the apparent inter-specific hybridisation events, chromosomal aberrations such as strain-specific insertions and deletions and loss-of-heterozygosity by gene conversion were also commonplace. While these events are likely to have affected many phenotypes across these strains, we have been able to link a specific deletion to the inability to utilise nitrate by some strains of *D. bruxellensis*, a phenotype that may have direct impacts in the ability for these strains to compete with *S. cerevisiae*.

## Author Summary

The yeast *D. bruxellensis* is of great importance in biofuel and fermented beverage industries, largely as a contaminant and/or spoilage organism. Its lifestyle is not unlike that of the wine/brewing/baking yeast *S. cerevisiae*, with independent evolutionary pathways having led to this convergence; these species are phylogenetically very distant. Unlike *S. cerevisiae*, *D. bruxellensis* is highly intractable in the laboratory; it is difficult to mate and to transform, making even the most basic genetic analysis very difficult. Thus we still have a great deal to learn about this economically important yeast. The latest gene sequencing technologies are, however, providing a means of addressing these limitations. The current manuscript describes a comparative genomics approach to providing insights into inter-strain variations that shape the genomic landscape of *D. bruxellensis*. Like other industrial yeasts, it has a diploid core genome, but there are also triploid isolates which possess the core diploid complement with an additional, more distantly related, full set of chromosomes. Evidence presented in this paper suggests that this form of triploidy has arisen more than once in the evolutionary history of *D. bruxellensis*, and it confers a selective advantage for strains of this yeast isolated from wineries.

## Introduction

*Dekkera (Brettanomyces) bruxellensis* has been described in the population ecology of various fermented beverages, such as wine, beer and cider [1]–[3], and is of increasing relevance to the biofuel industry [4]. Recent genomic sequencing of this species is beginning to reveal the mechanisms by which it is able to survive the harsh environment of alcoholic fermentation, primarily through gene-family expansions in membrane transporters and oxidoreductase enzyme classes that are predicted to facilitate nutrient scavenging and maintain redox homeostasis respectively [5]. However, our understanding of how other industrially important traits have evolved in *D. bruxellensis* lags well behind what is known for *S. cerevisiae* [6].

In general, *D. bruxellensis* utilises a make-accumulate-consume strategy similar to that found in *S. cerevisiae* [7], however traits, including carbon and nitrogen source utilisation [8], vary considerably between *D. bruxellensis* strains. For example, it was recently shown that nitrate utilisation enables *D. bruxellensis* to out-compete *S. cerevisiae* in continuous industrial fermentations [9], and key genes involved in nitrate assimilation were found in a cluster in the partial genome sequence of *D. bruxellensis* strain CBS2499 [10]. Nonetheless, nitrate utilisation is not a defining feature of this species. Nearly one third of *D. bruxellensis* isolates from a range of sources do not grow on nitrate as a sole nitrogen source [8]; presumably nitrate assimilation is less important for *D. bruxellensis* in some fermentation ecosystems. In another recent study, variation in sulphite tolerance in *D. bruxellensis* was linked to amplified fragment length polymorphism (AFLP) and 26S rDNA genetic markers [11], inferring a genetic basis for previously reported regional variation and groupings of this yeast across Australian wineries [1].

To date, *de-novo* assemblies exist for genomes of two *D. bruxellensis* wine isolates, AWRI1499 [5] and CBS2499 [12], from Australia and France, respectively. Unlike AWRI1499, CBS2499 has the same 26S rDNA sequence as the *D. bruxellensis* type strain CBS 74 (unpublished data), a lambic-beer isolate. This data, in combination with AFLP genotyping [1], infers the two sequenced strains are likely to be highly divergent. AWRI1499, a representative isolate of a sulphite-tolerant genotype group [11], is an allotriploid comprising a moderately heterozygous diploid, and divergent haploid complements [5]. Thus far it remains unclear to what degree intra-specific differences observed using methods such as AFLP may simply reflect presence or absence of all or part of the divergent haploid genome found in AWRI1499. CBS2499 was assembled as a pseudo-haploid [12], preventing such comparison with other *de-novo* assemblies.

To improve our understanding of genome diversity amongst *D. bruxellensis* wine isolates and gain insights into the evolution of industrially relevant traits in this important microorganism, we have performed mapping-assemblies of CBS2499 and two newly sequenced Australian *D. bruxellensis* wine strains against the reference genome sequence of AWRI1499. Comparative genomics of the four strains reveals that presence of a divergent haploid genome is not a feature restricted to AWRI1499, but has arisen through at least two independent ‘hybridisation’ events. In addition to large-scale ploidy variation, gene conversion and allelic expansion appear to be key molecular mechanisms driving strain divergence. Some phenotypes, such as nitrate/nitrite utilisation, on the other hand, are determined by genomic insertions and deletions (InDels).

## Results

### Analysis of Dekkera genomes

In order to compare the genomic complements of *D. bruxellensis* strains, a re-sequencing strategy was used to align genome data from short-read sequencing (2×100 bp) for three strains against the published draft genome assembly of *D. bruxellensis* strain AWRI1499 [5]. Two of the assemblies were for strains sequenced specifically for this work; AWRI1608 and AWRI1613. For the third, CBS2499, comparable data (2×100 bp format genome data) used as part of the *D. bruxellensis* CBS2499 draft genome assembly [12] was obtained from the NCBI short read archive. The two newly sequenced strains were chosen because they have divergent AFLP genotypes and, with AWRI1499 represent 98% of *D. bruxellensis* isolates associated with Australian wineries [1]. In all, the divergence between all four strains, as determined from AFLP analysis, is considerable and therefore should provide insights into the genomic landscape of wine isolates of this important yeast.

**Figure 1:**
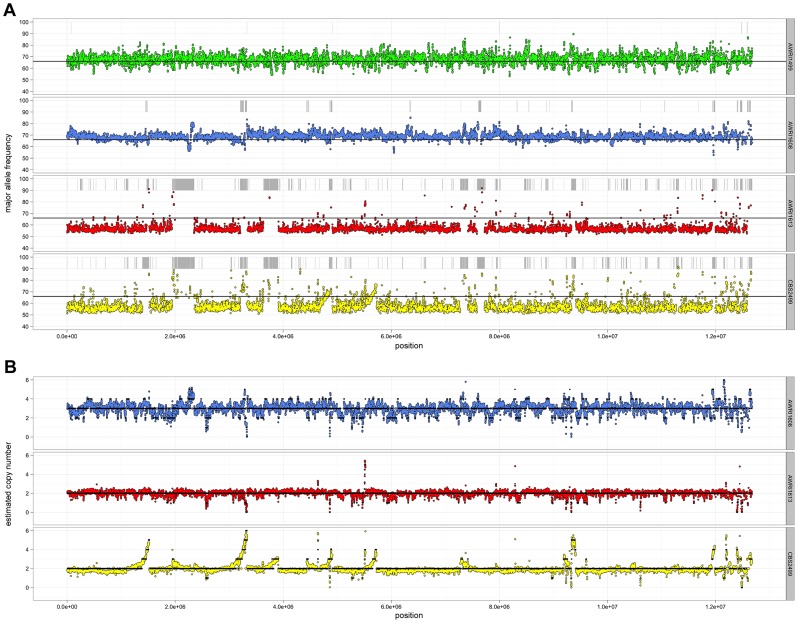
Resequencing analysis of *D. bruxellensis* isolates. (A) Single nucleotide polymorphism analysis. For each strain, heterozygous nucleotides were identified and the proportion of aligned reads containing each of the variant bases recorded. The average major allele frequency was then calculated for sliding windows across the genome (5 kb window, 1 kb step) and plotted central to each window. Any regions that lacked heterozygous bases were classified as regions of loss-of-heterozygosity (LOH) and are indicated by grey bars above each plot. The solid black line represents a major allele frequency of 0.66 that would be expected for heterozygous a triploid genome. (B) Copy number variation analysis. For each strain, the average sequencing read depth was recorded for sliding windows across the genome (5 kb window, 1 kb step) and are presented relative to a predicted triploid state for AWRI1608 and a diploid state for AWRI613 and CBS2499. Solid black lines indicate proposed ploidy levels across the genome based on segmental smoothing (see materials and methods).

Given the unusual nature of the *D. bruxellensis* AWRI1499 genome (triploid hybrid comprised of a closely related diploid set of alleles and a third distantly related genomic complement), it was of interest to determine whether this genomic organisation is a defining characteristic of this species. Sequence alignments were therefore interrogated globally to determine genomic ploidy and the levels of both inter- and intra-allelic genetic diversity (Fig. 1, Datasets S1 and S2).

Ploidy levels across the genomes were estimated by taking advantage of allele proportions. In a triploid genome, it is expected that the maximum average frequency of a particular allele at a heterozygous site will be approximately 0.66 (due to a base difference in a single allele), while this number will be closer to 0.5 for heterozygous sites in a diploid. The observed average major allele frequency was therefore calculated across the entire genome of each isolate using a sliding window approach (Fig. 1A). As a triploid control, RNA-seq data for AWRI1499 was mapped to the AWRI1499 genome and this showed a maximum average allele proportion consistent with its triploid state (0.68±0.04, data not shown). AWRI1608 also displayed an average allele proportion (0.69±0.03), consistent with this isolate being triploid. However, both CBS2499 (0.58±0.05) and AWRI1613 (0.57±0.03) displayed maximum allele frequencies consistent with these isolates being diploid.

**Figure 2:**
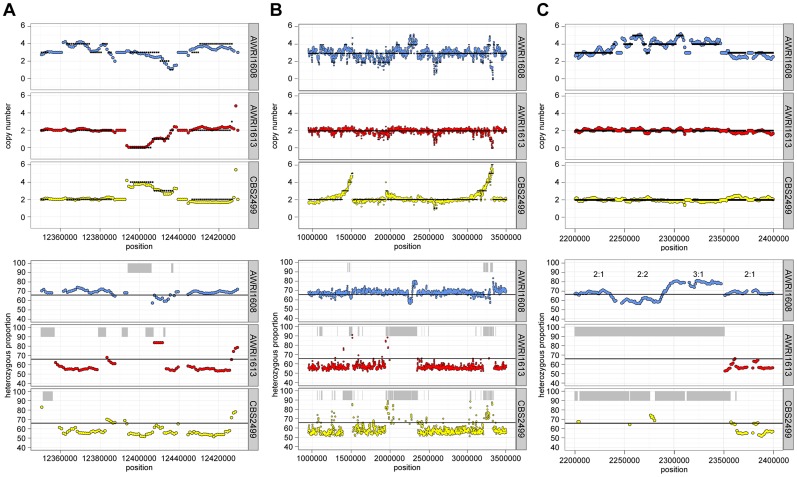
Inter-strain genetic variation at discrete genomic loci. Data is presented for three distinct genomic loci (A–C). Estimates of copy number and heterozygous allele frequencies were calculated and are presented as in Fig. 1.

For all strains, while the average maximum allele frequency approximated to either 0.66 or 0.5 there were many localised regions that differed from these values, including significant portions of the genomes that displayed loss of heterozygosity (LOH; 17.9% of AWRI1613, 16.3% of CBS2499 and 3% of AWRI1608) (Fig. 1A, Fig. 2). As these differences in local allele proportions may be due to copy number variation (CNV), such as heterozygous deletions or genomic duplications, CNV was also determined globally for each of the genomes (Fig. 1B). While copy number was relatively stable, there were many instances of localised copy number variation in each strain, and of opposing copy number changes in the same genomic region between strains (Fig. 2A). Copy number amplification was especially prominent in CBS2499 with several genomic regions displaying effective copy numbers of 4 n or greater (Fig. 1B, Fig. 2B). Regions of increased copy number in CBS2499 appear to coincide with the ends of genomic scaffolds in both the AWRI1499 and CBS2499 [12] assemblies (Fig. S1), a feature not described in the CBS2499 *de-novo* assembly. This may be indicative of sub-telomeric amplification of sequences, which is common to other yeasts including *S. cerevisiae* and *Cryptococcus neoformans* [13], [14]. Examination of the functional annotation for the genes in these expanded regions revealed a statistically significant enrichment for those encoding proteins involved in carbohydrate metabolic processes (p = 6.5×^10-7^) and may therefore indicate adaptation to utilisation of specific carbon sources by this strain.

It was also apparent that there was co-localisation of copy number variation and alterations in allele frequencies (including the majority of LOH events). This is consistent with gene conversion, rather than heterozygous deletion, being responsible for the majority (95%) of genomic regions displaying LOH across the strains. For example, Fig. 2C describes a 100 kb genomic locus in which loss of heterozygosity has occurred in AWRI1613 and CBS2499 without altering the normal diploid genomic complement of these strains, while in AWRI1608, the otherwise triploid state is predicted to have been amplified to a tetraploid complement. Interestingly, this amplification in AWRI1608 is complicated by the fact that the allelic ratios change from 2∶2 to 3∶1 within the amplified region. This change in allelic ratios is indicative of either gene conversion of one allele following amplification of the region, or two amplification events that each amplified adjacent parts of the region carrying different homologs.

### Allelic relationship across Dekkera isolates

**Figure 3:**
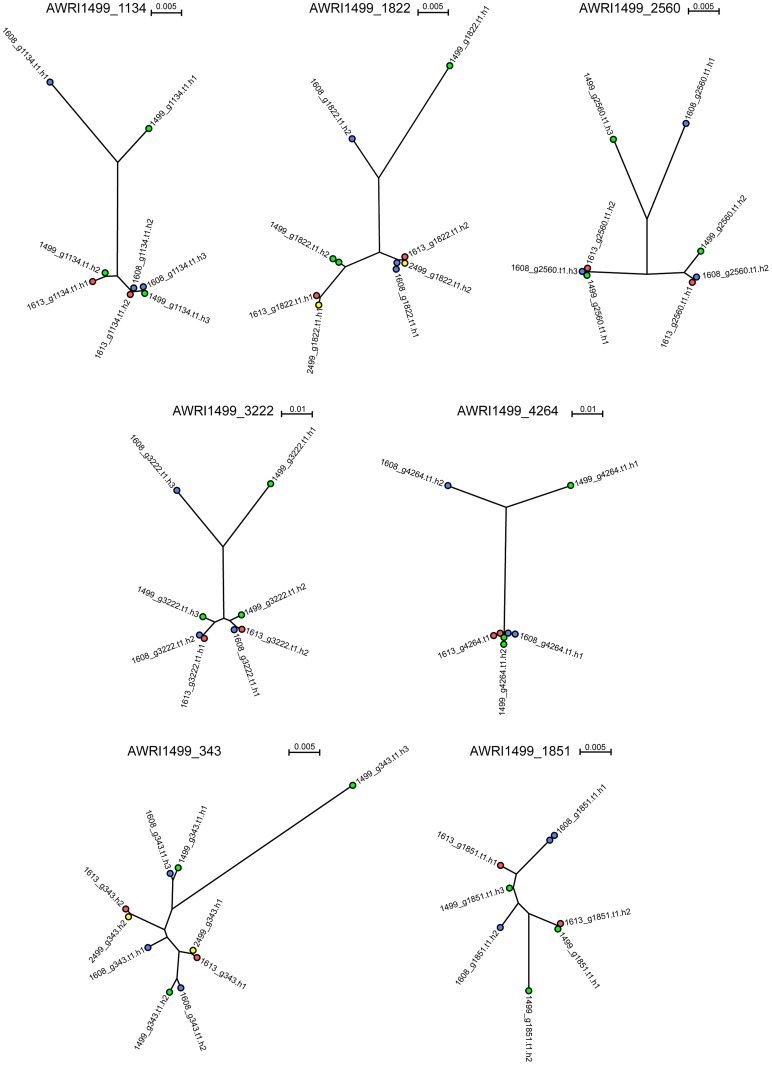
Haplotype analysis of *D. bruxellensis* isolates. Distinct haplotypes were assembled for conserved open reading frames and subjected to maximum-likelihood phylogenetic analysis [15], [16]. Nodes are color-coded according to strain AWRI1499 (green), AWRI1608 (blue), AWRI1613 (red), CBS2499 (yellow). Nodes for CBS2499 are only shown where haplotypes were different to those of AWRI1613.

It has been shown previously that the allotriploid genome of AWRI1499 consists of two highly related sets of chromosomes in addition to a third, more distantly related, set [5]. As the genomic analyses of AWRI1608, AWRI1613 and CBS2499 predicted only one of these strains to also be a triploid, it was of interest to determine the relationship between each of the haplotypes across all of the strains in order to ascertain whether either of the diploid strains contained the “divergent” haplotype of AWRI1499. Seven loci that displayed three clearly defined haplotypes in AWRI1499 [5] were selected with individual haplotypes derived for each locus in each strain by taking advantage of co-occurring SNPs within individual reads. This resulted in a total of ten possible haploid sequences (3+3+2+2) for each locus for which maximum-likelihood phylogenies [15], [16] were constructed (Fig. 3, Dataset S3). Consistent with whole genome alignments (Fig. S2), AWRI1613 and CBS2499 alleles were identical for five of seven loci, and exhibited only minor differences for the remaining two (AWRI1499_1134 and AWRI1499_1822). Furthermore, in the majority of cases, the phylogeny was resolved into a relationship whereby two of the alleles from AWRI1499 and AWRI1608 and both alleles from AWRI1613 and CBS2499 formed a highly related clade, while the third alleles from AWRI1499 and AWRI1608 were both divergent from this conserved clade and also distinct from one another. For the remaining two loci, it appears likely that gene conversion has resulted in either one or both of these divergent alleles being replaced by an allele that is consistent with the conserved clade.

Maximum likelihood phylogenies for individual haplotypes derived at five additional genomic loci (Fig. S3, Dataset S3), previously sequenced for a collection of international *D. bruxellensis* strains [17], revealed similar topologies. Together with a 26S rDNA phylogeny (Fig. S4), these data provide evidence that predominant Australian *D. bruxellensis* strain AWRI1499 [1] is similar to South African wine-related *D. bruxellensis* strains CBS4481 (Y900) and CBS5206 (Y908). Of note, the *DbHAD* phylogeny (Fig. S3E) suggested that the horizontal transfer of an adenyl deaminase from an unknown Proteobacterial species [10] occurred prior to divergence of the AWRI1499 and AWRI1608 ‘third haplotype’ donors. A protein-based phylogeny (Fig. S5) suggests that *DbHAD1* may have descended vertically from the common progenitor of *D. bruxellensis* and *Ogataea parapolymorpha*.

**Figure 4:**
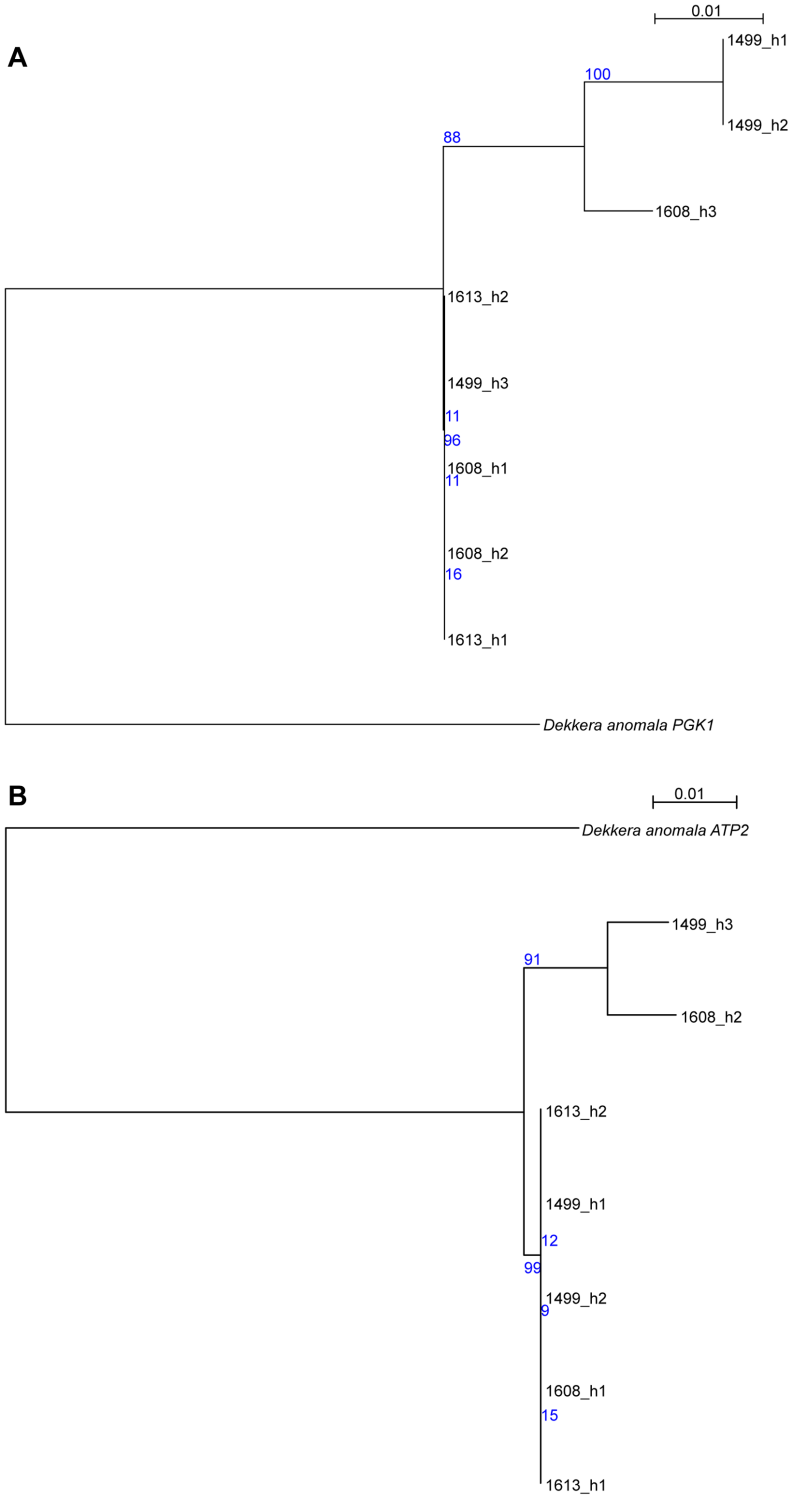
Comparison of *D. anomala* and *D. bruxellensis* gene sequences. Haplotypes from regions of *D. bruxellensis* AWRI1499, AWRI1608 and AWRI1613 mapping assemblies corresponding to (A) *D. anomala* CBS8139 gene *PGK1* (genbank accession KF042711.1) and (B) *D. anomala* CBS8139 gene *ATP2* (genbank accession KF042617.1) were aligned and subjected to maximum-likelihood phylogenetic analysis [15], [16], with bootstrap support from 1000 randomisations indicated in blue.

While the initial analysis of the AWRI1499 genome failed to identify a potential ‘donor’ species for the divergent alleles [5], we sought to determine if there was sequence data now available that would shed new light on this. Protein-based maximum-likelihood phylogenies were therefore produced for each of the haplotype groups from *D. bruxellensis* for three of the open reading frames (ORFs) presented in Fig. 3, in addition to homologs identified in the Genbank non-redundant protein database (Fig. S6). This analysis clearly shows all of the *D. bruxellensis* alleles to be far more closely related to each other than to any other available protein sequences. A small number of gene sequences available for *D. anomala*, the closest known relative of *D. bruxellensis* according to 26S rDNA based phylogenies [18], were then used as nucleotide queries against the AWRI1499 blast database. Two accessions, annotated as *ATP2* and *PGK1,* were strong positive matches to AWRI1499 open reading frames, with 92% and 93% nucleotide identity. Nucleotide-based maximum-likelihood phylogenies for these ORFs, with haplotypes extracted from AWRI1499, 1608 and 1613, were performed (Fig. 4). The *D. anomala* sequences were not closely related to any of the *D. bruxellensis* haplotypes. As such, the potential source of the divergent alleles in the triploid strains remains to be determined.

### Variation in nitrate assimilation potential

While there was significant variation in SNP diversity across strains, strain-specific genomic deletions were found to be far less common, with an average of only 0.15% of the genome lost, across the three strains relative to AWRI1499. The majority (97%) of these deletions were found in AWRI1613.

**Figure 5:**
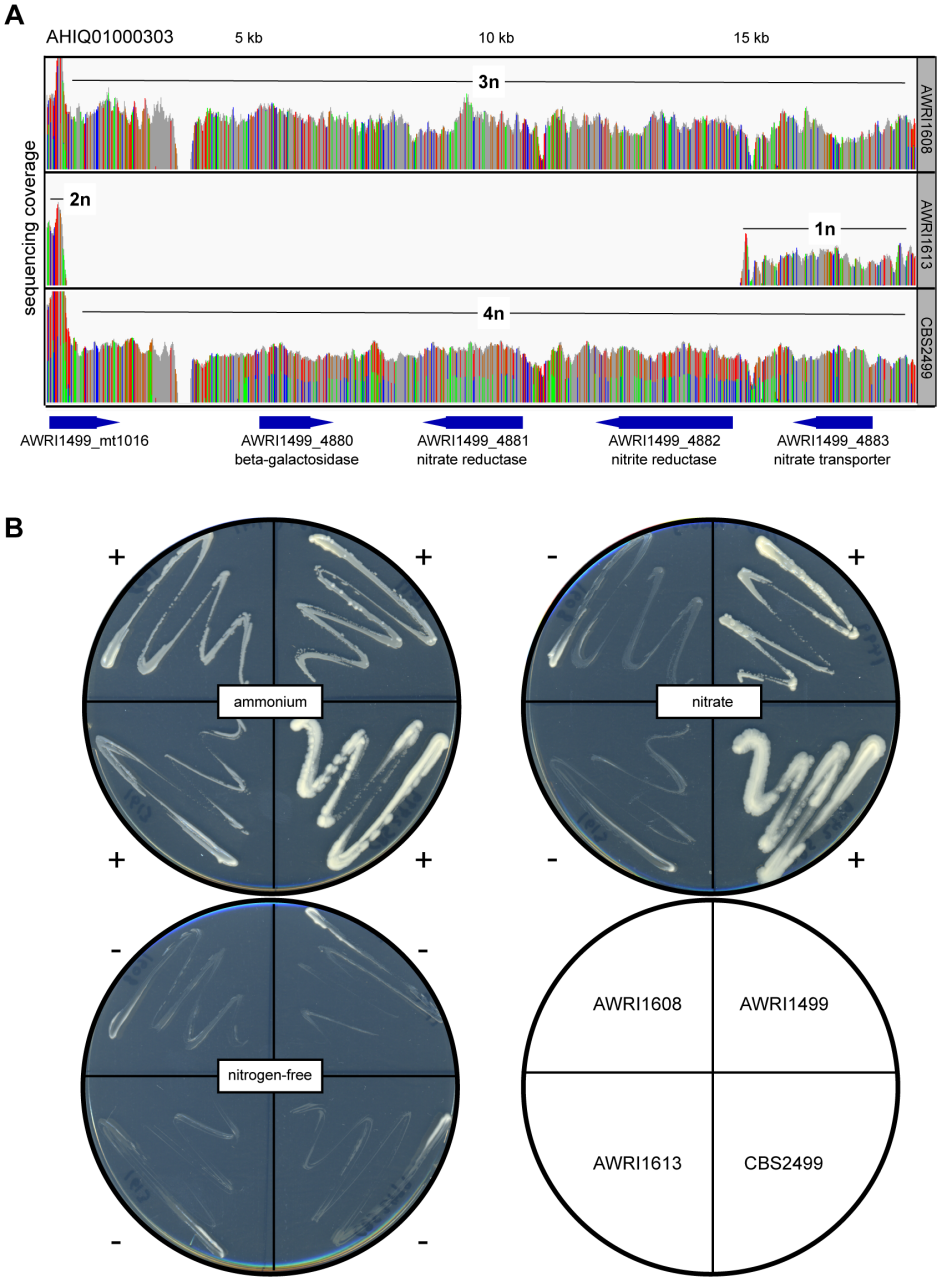
Loss of nitrate and nitrite assimilation in *D. bruxellensis* strains. (A) Sequencing coverage across AWRI1608, AWRI1613 and CBS2499. Bases that are in disagreement to the AWRI1499 reference strain are colored according to their sequence and proportion at that position (AWRI1499, green; AWRI1608, blue; AWRI1613, red). The positions of open reading frames in this region (according to the AWRI1499 genome annotation) are also shown. (B) Phenotypic analysis of *D. bruxellensis* strains growing on either on ammonium and nitrate. Strains scored as showing either positive (+) or negative (-) growth are indicated.

Of the genomic loci that did display strain-specific deletions, one region that was lost specifically in AWRI1613 was of particular interest as it involved the *D. bruxellensis* nitrate assimilation cluster (Fig. 5). While nitrate utilisation is common throughout *Ascomycota*, genes associated with the nitrate assimilation cluster are generally confined to *Pezizomycotina*; *Dekkera*, *Ogataea*, *Wickerhamomyces* and *Blastobotrys* are the only genera within the *Saccharomycotina* where this cluster has been identified. In these species the nitrate cluster appears to have been retained from the last common ancestor with the *Pezizomycotina* (Fig. S7). However, despite this ancient evolutionary conservation, it is apparent that both the nitrate and nitrite reductase genes have been lost in AWRI1613, along with an adjacent *β*-galactosidase gene. Furthermore, while this cluster is present in AWRI1608 it is predicted to have undergone LOH via gene conversion, resulting in three identical alleles. In contrast, the nitrate cluster of CBS2499 is predicted to have undergone a duplication event resulting in four copies of this genomic region being present in a 1∶1 ratio of two alleles (Fig 2A, Fig 5A).

Consistent with presence or absence of ORFs encoding these key assimilatory enzymes, AWRI1499 and CBS2499 displayed robust growth on nitrate as a sole nitrogen source whereas AWRI1613 was unable to grow on this medium (Fig. 5B). Interestingly, despite the presence of the nitrate assimilation locus, AWRI1608 was also unable to utilise nitrate. In order to determine if this loss of nitrate utilisation was due to frameshift or nonsense mutations in the homozygous coding sequences of the AWRI1608 cluster, the ORFs of the nitrate and nitrite reductases were compared to haplotyped sequences from CBS2499 (Dataset S4). In each case, there were no obvious truncated coding regions or non-synonymous mutations present in the AWRI1608 ORFs, compared to either of the CBS2499 haplotypes that would be expected to cause the drastic changes to enzyme function that could account for the loss of nitrate utilisation in AWRI1608.

To further investigate the cause of the non-nitrate utilisation phenotype of AWRI1608, a high-affinity nitrate transporter gene that lies adjacent to the two reductases and the genomic region surrounding the two putative nitrate assimilation transcription factors of *D. bruxellensis* [10] was also investigated in these strains. The gene encoding the nitrate transporter was shown to be homozygous in both strains that could not grow on nitrate (AWRI1608 and AWRI1613) but was heterozygous in AWRI1499 and CBS2499 (data not shown). However, even in the two homozygous strains the ORF is predicted to encode a full-length, functional, nitrate transporter. Similarly, the genomic region encompassing both putative regulatory proteins was predicted to be present in all four strains; as for the nitrate and nitrite reductase genes in AWRI1608, both ORFs displayed LOH in AWRI1608, AWRI1613 and CBS2499 (data not shown). In contrast, this region was shown to be heterozygous in AWRI1499.

## Discussion

**Figure 6:**
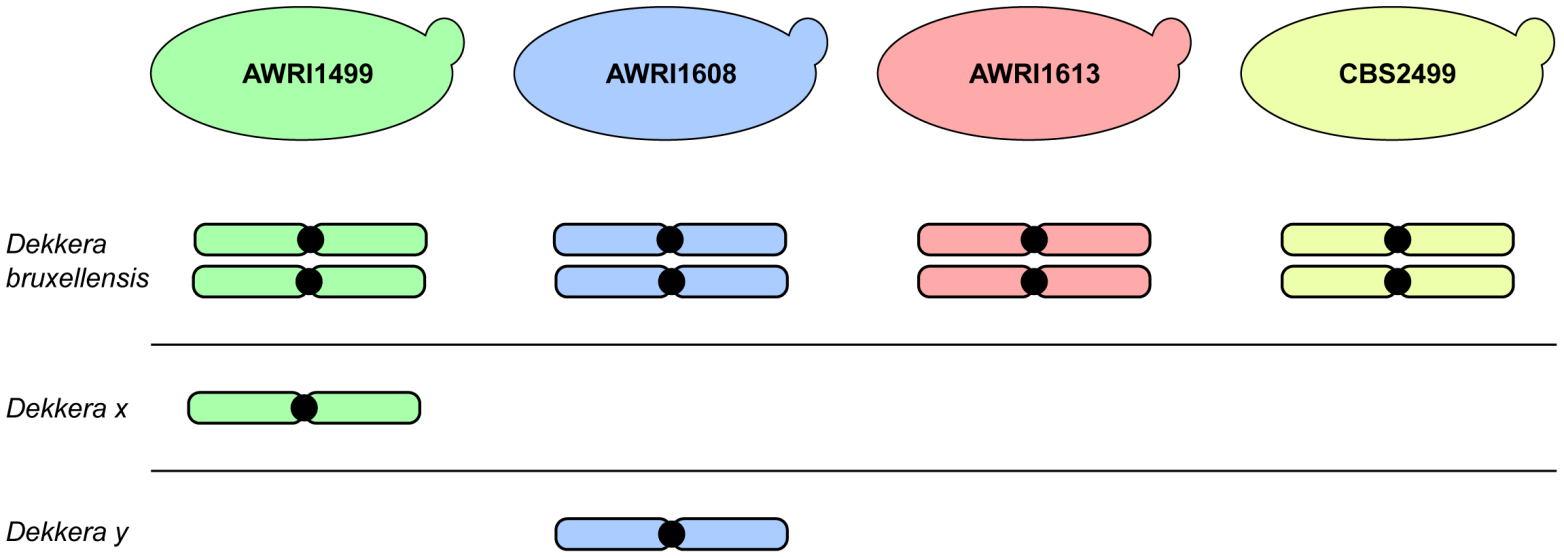
Schematic representation of *D. bruxellensis* strain genomes. Each of the *D. bruxellensis* strains is predicted to contain a conserved diploid set of chromosomes. In addition, AWRI1499 and AWRI1608 are predicted to both contain a third full set of chromosomes that have been inherited from more distantly related strains or a closely related species that is unique to each strain (*Dekkera x* and *Dekkera y*).

The advent of next generation sequencing has enabled a significant increase in knowledge regarding the genomic makeup of important, but often genetically intractable, industrial yeasts. This manuscript describes the first analysis of inter-strain variation in the genomic landscape of *D. bruxellensis*. The most prominent finding of these genome comparisons was the common occurrence of triploid hybrids in the strains examined. AWRI1613 and CBS2499 were determined to be diploid in this study, with each strain possessing a pair of closely related chromosomes with moderate levels of heterozygosity. However, AWRI1499 and AWRI1608, the most common strains found in Australian wineries, were shown to be triploid. While triploid, both AWRI1499 and AWRI1608 contain pairs of chromosomes that are closely related to those found in the diploid strains suggesting that a diploid complement comprises the basis of the *D. bruxellensis* genome. The third, complete, set of more distantly related chromosomes present in AWRI1499 and AWRI1608 are therefore likely to have been introduced via hybridisation with a distantly related strain of *D. bruxellensis* or possibly another closely related but as yet undescribed species (Fig. 6). Furthermore, the divergent third sets of chromosomes present in each triploid are, in-turn, distantly related to each other, indicating that the two triploid strains likely arose from independent hybridisation events.

The results of this genomic study therefore suggest that the *D. bruxellensis* genomic landscape is similar to counterparts in the *Saccharomyces sensu stricto* clade, where inter-specific hybrids between *S. cerevisiae, S. kudriavzevii*, *S. uvarum* and *S. eubayanus* can be found in natural environments and in industrial fermentations [19]–[22]. Furthermore, *Saccharomyces spp*. interspecific hybrids are often allotriploid, with a ‘diploid’ complement coming from *S. cerevisiae* and a ‘haploid’ input from a non-*cerevisiae* parent. These *Saccharomyces sensu stricto* hybrids have been isolated from cold winemaking and brewing environments, where it is suggested the hybrid has a selective advantage over its parents. In these situations, the *S. cerevisiae* genomic component provides the means to efficiently ferment sugar to ethanol while genomic contributions from cold tolerant *Saccharomyces spp*. allow the hybrid strains to ferment at temperatures that are normally too low for *S. cerevisiae* [6], [19], [23]. In fact, strains of *S. pastorianus*, the yeast species responsible for the vast majority of lager beer fermentations, are hybrids generated from matings between *S. cerevisiae* and *Saccharomyces eubayanus*. At least one line of these hybrids has allotriploid origins, as was observed for the two *D. bruxellensis* hybrids analysed in this work [19], [20], [24].

At this time it is not possible to determine whether the ‘additional haploid’ inputs in the karyotypes of the two triploid *D. bruxellensis* strains described in this paper originate from one or more non-*D. bruxellensis* species or distantly related *D. bruxellensis* strains. However, as for lager brewing, it appears that the formation of these triploid hybrid strains may have resulted in a population replacement event, with the hybrid strains representing 92% of isolates from across the Australian wine industry. Based upon a limited multi-locus analysis, some previously analysed international strains [17] bear resemblance to AWRI1499 and AWRI1608, therefore it is possible that the current population structure in Australian wineries reflects historical gene flows and bottlenecks. It remains to be determined whether the additional sets of chromosomes in AWRI1499 and AWRI1608 confer a selective advantage in the winery environment, although increased levels of resistance to sulphite, the primary means of *D. bruxellensis* control in a winery setting, may be at least partially responsible [11].

As for presumptive selective pressures underpinning hybrid prevalence, the driver for loss of nitrate assimilation ability in specific strains of *D. bruxellensis* remains unclear. Ammonium is fully utilised by *S. cerevisiae* and other wine yeast species during alcoholic fermentation [25]. However *D. bruxellensis* appears to be the only wine yeast species that can assimilate nitrate, which is reported to be at levels of between 0.9 and 53.7 mg/l in Californian wine [26]. One might predict therefore that, in ecological settings where nitrate is available and other nitrogen sources are limited, nitrate assimilation would provide *D. bruxellensis* with a selective advantage. Yet up to a third of *D. bruxellensis* wine isolates fail to grow on nitrate [8]. Interestingly, it was recently shown that during anaerobic fermentation nitrate assimilation in *D. bruxellensis* favours the production of acetic acid over ethanol while partially abolishing the Custers effect [27]. This impact of nitrate assimilation may be detrimental in some environmental settings, thereby providing a selective pressure for its loss.

This phenomenon of loss of nutrient utilisation is not unknown in nature. For example, there is a concerted loss of the galactose (GAL) catabolism cluster in Japanese isolates of *Saccharomyces kudriavzevii* when compared to European relatives. In this example, the Japanese strains show degeneration of the genes involved in the utilization of galactose to pseudogene equivalents, while this cluster is completely active in European strains [28]. Evidence was also presented for a selective pressure driving loss of function for all members of the GAL pathway thereby producing a GAL^−^ phenotype, as the presence of partial function in the pathways were suggested to have fitness costs.

At face value, this does not appear to be the case for loss of nitrate assimilation in AWRI1613, which still retains the coding region for the nitrate transporter. However, it is currently not known whether the presence of this transporter may be due to pleiotropy; it may, for example, be required for secondary transport functions. It is also possible that it may be non-functional, although given that the nucleotide sequence of the nitrate transporter ORF in AWRI1613 represents the opposite haplotype group to the homozygous transporter sequence from AWRI1608 (Fig. S8), it would be expected that at least one of these strains would have a functional transporter as evidenced by the nitrate assimilation phenotype of CBS2499, which has one copy of each haplotype.

Further study of nitrate assimilation in *D. bruxellensis* will reveal the molecular mechanisms driving the phenotype towards, or away from, utilisation of this nitrogen source, augmenting knowledge gained through detailed studies of the preferred yeast model system for nitrate assimilation, *Ogataea parapolymorpha* [29].

## Materials and Methods

### Yeast strain, nucleic acid preparation, and sequencing

D. bruxellensis strains AWRI1608 and AWRI1613 were obtained from The Australian Wine Research Institute Microorganisms Culture Collection. For nitrate assimilation tests, strains were grown on solid YPD for 2 days at 30°C, then plated onto either YNB+ nitrate, YNB+ ammonium as a positive control, or YNB with no nitrogen source as a negative control. Plates were then incubated for 7 days at 30°C.

Genomic DNA was prepared using a standard zymolyase and phenol-chloroform extraction from cultures grown under standard conditions. DNA sequencing was performed using 2×100 bp paired-end chemistry on the Illumina HiSeq2000 (Ramaciotti Centre, Sydney Australia).

AWRI1499 genome sequences were obtained from Genbank (Accession number AHIQ0100000). CBS2499 short-read sequences were obtained from the NCBI short-read archive (Accession number SRR065689). Sequence data for AWRI1608 and AWRI1613 have been deposited in the NCBI short-read archive under the Bioproject accession PRJNA213658. 26S rDNA sequences for AWRI1499, 1608 and 1613 have been deposited with GenBank (accessions KF781196, KF781197 and KF781198, respectively).

### Mapping assemblies and analysis

Short read sequences were mapped to the AWRI1499 genome using Novoalign v2.08.01 (www.novocraft.com). The .sam files produced by Novoalign (default parameters; -F ILM1.8 –ILQ_SKIP -i PE 100-1000 -o SAM) were converted to sorted .bam files using samtools *view* v0.1.18 [30]. SNPs, and regions of LOH and gain-of-heterozygosity (GOH) were identified from the alignments using the *pileup2snp* functionality of Varscan v2.3 (default parameters; -min-coverage 10) [31] combined with custom python scripts and presented relative to a concatenated AWRI1499 genome sequence. The position of individual AWRI1499 Genbank contigs and ORF annotations within the concatenated genome sequence are provided in Datasets S5 and S6 respectively. Sequence alignments were visualized using the Integrated Genome Browser v2.0 [32]. Any region displaying a maximum allele frequency of >95% was classed as being homozygous for that allele.

Sequencing coverage was extracted from alignments in .bam format using *mpileup* from the samtools v0.1.18 package [30] with actual coverage values converted to changed ploidy levels in sliding windows using custom python scripts. Segmental smoothing of copy number alterations calculated final copy number based on rounding the average value across 21 adjacent genomic windows. To provide additional robustness against single outliers producing small false-positive intervals of altered ploidy levels, a difference of ±0.75 was required between the average ploidy of the current 21 window genomic segment and the predicted ploidy level of the previous genomic segment in order to trigger a change in the final predicted ploidy level. If this threshold was not met, the average value obtained for the 21 segment window was rounded to the ploidy level of the previous genomic segment.

Phylogenies were constructed using PhyML v3.0 (GTR model; default parameters) and visualized using Seaview v4.0 [15], [16].

## Supporting Information

Figure S1 Copy number variation in CBS2499. (**A**) Copy number variant estimation. Average coverage was calculated from a .bam file using samtools mpileup (v. 0.1.18) and custom scripts using a window of 5 kb and a step of 1 kb. (**B**) Relative read coverage calculated using the count function of igv tools and displayed using IGV (v. 2.3.20). Maximum coverage was set at six for direct comparison to panel A. For both datasets, short read-sequence data from CBS2499 (NCBI SRA accession SRR065689) was mapped against the CBS2499 genome (http://genome.jgi.doe.gov/Dekbr2) using Novoalign (v3.01.00).
(PDF)

Figure S2 Whole genome phylogenies of *D*. bruxellensis. Whole-genome alignments were produced for each strain by converting nucleotides within the AWRI1499 reference based upon the results of the read-mapping and SNP analysis. Any regions displaying nucleotide insertions or deletions or low read coverage (<10 reads) in at least one strain were then removed prior to further analysis. The maximum-likelihood phylogeny was then calculated for these alignments using PhyML.
(TIF)

Figure S3 Haplotype analysis for additional *D. bruxellensis* isolates. Distinct haplotypes were assembled for genomic regions of AWRI1499, AWRI1608, AWRI1613 and CBS2499 that matched loci studied by Hellborg and Piškur [17] (data downloaded from NCBI for additional strains on 21 October 2013). Haplotype sequences (Dataset S3) for (**A**) *DbYER090*, (**B**) *DbYDR513*, (**C**) *DbYLR048Y*, (**D**) *DbYDL040* and (**E**) *DbHAD1* were subjected to maximum-likelihood phylogenetic analysis [15], [16]. Nodes are color-coded according to strain AWRI1499 (green), AWRI1608 (blue), AWRI1613 (red), CBS2499 (yellow).
(PDF)

Figure S4 Phylogenic relationship of *D. bruxellensis* isolates. 26S rDNA (D1/D2 domain) sequences for AWRI1499, AWRI1608 and AWRI1613 [1] and 30 other *D. bruxellensis* isolates [17] were aligned and a neighbor-joining tree constructed after removal of all gapped bases [15], with *D. anomala* as the outgroup. Bootstrap support from 1000 randomisations indicated in blue. Isolates exhibiting more than one ribotype denoted by different letters.
(PDF)

Figure S5 Phylogenic analysis of horizontally transferred adenyl deaminase. Maximum-likelihood phylogenies were produced for haplotype-resolved predicted protein sequences of *DbHAD1* for multiple *D. bruxellensis* isolates [17], in addition to the best matches present in the Genbank non-redundant protein database (at 21 October 2013) and the canonical *D. bruxellensis* adenine deaminase (*DbADE1*) [10]. *Escherichia coli* adenosine deaminase was used as the outgroup, and bootstrap support from 1000 randomisations is indicated in blue.
(PDF)

Figure S6 Broader phylogenic analysis of *D. bruxellensis* proteins. Maximum-likelihood phylogenies were produced for haplotype-resolved predicted protein sequences of three *D. bruxellensis* ORFs in addition to the best matches present in the Genbank non-redundant protein database.
(PDF)

Figure S7 Phylogenic analysis of the nitrate assimilation cluster in *D. bruxellensis*. Maximum-likelihood phylogenies were prepared separately for both nitrite (**A**) and nitrate (**B**) reductases. All homologous protein sequences from the nr Genbank dataset for members of the *Saccharomycotina* subphylum are included, in addition to representative sequences from both *Pezizomycotina* and *Basidiomycota*. Sequences from *Mucor circinelloides* were included as an outgroup.
(PDF)

Figure S8 Phylogenic analysis of the predicted nitrate transporter of *D. bruxellensis*. A maximum-likelihood phylogeny was constructed from the nucleotide sequence of haplotype-resolved ORFs from AWRI1499, AWRI1608, AWRI1613 and CBS2499. Node colors represent the ability of the parent strain to utilize nitrate as a nitrogen source (green - growth; red - no growth).
(PDF)

Dataset S1 Calculated read coverage and estimated ploidy levels across D. bruxellensis strains. Observed coverage was calculated from a .bam file using samtools mpileup (v. 0.1.18) and custom scripts using a window of 5 kb and a step of 1 kb, with “average coverage” calculated as the average coverage across all windows. “Genomic position” references the centre of each window.
(XLSX)

Dataset S2 Heterozygosity and allelic proportions across *D. bruxellensis* strains. For each strain, heterozygous nucleotides were identified and the proportion of aligned reads containing each of the variant bases recorded. The average major allele frequency was then calculated (heterozygous_proportion) for sliding windows across the genome (5 kb window, 1 kb step) and recorded central to each window (genomic_position).
(XLSX)

Dataset S3 Haplotype-resolved nucleotide sequences for a selection of *D. bruxellensis* ORFs. Sequences are presented in fasta format.
(TXT)

Dataset S4 Protein-based alignments of the nitrate reductase cluster. Clustal alignments were produced for the predicted nitrate reductase (*YNR1*), nitrite reductase (*YNI1*) and nitrate transporter (*YNT1*) proteins from AWRI1608 and CBS2499.
(DOCX)

Dataset S5 Revised annotation of the AWRI1499 genome. Data is presented in general feature format (.gff).
(TXT)

Dataset S6 Annotation of AWRI1499 concatenated genome sequence. The start coordinates of each of the AWRI1499 contigs (listed by Genbank accession) within the concatenated sequence.
(XLSX)
