# Supplementary figures and images for: Insights into the Dekkera bruxellensis Genomic Landscape: Comparative Genomics Reveals Variations in Ploidy and Nutrient Utilisation Potential amongst Wine Isolates
Source: PLoS Genet. 2014 Feb 13;10(2):e1004161. doi: 10.1371/journal.pgen.1004161 (PMC3923673; doi:10.1371/journal.pgen.1004161)

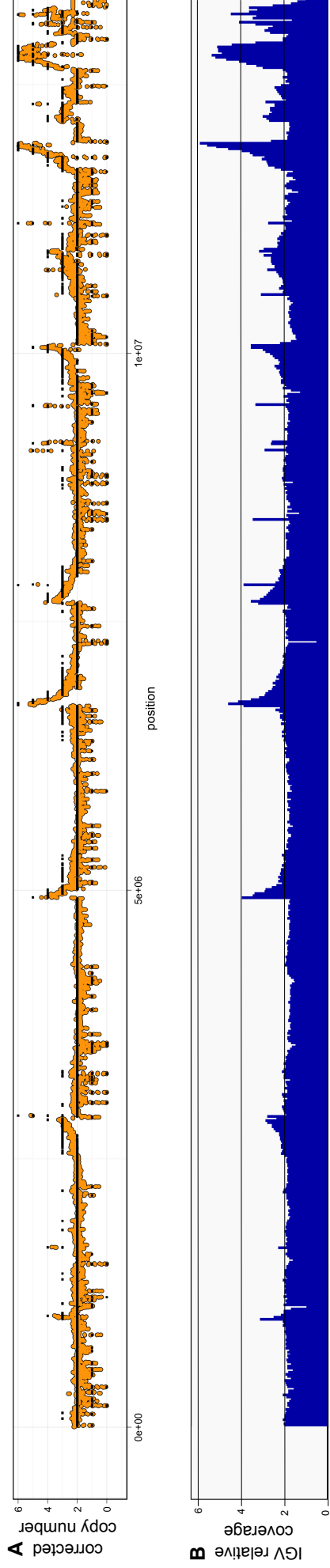

Supplement: Figure S1 — Copy number variation in CBS2499. (A) Copy number variant estimation. Average coverage was calculated from a .bam file using samtools mpileup (v. 0.1.18) and custom scripts using a window of 5 kb and a step of 1 kb. (B) Relative read coverage calculated using the count function of igv tools and displayed using IGV (v. 2.3.20). Maximum coverage was set at six for direct comparison to panel A. For both datasets, short read-sequence data from CBS2499 (NCBI SRA accession SRR065689) was mapped against the CBS2499 genome (http://genome.jgi.doe.gov/Dekbr2) using Novoalign (v3.01.00). (PDF) [file pgen.1004161.s001.pdf]

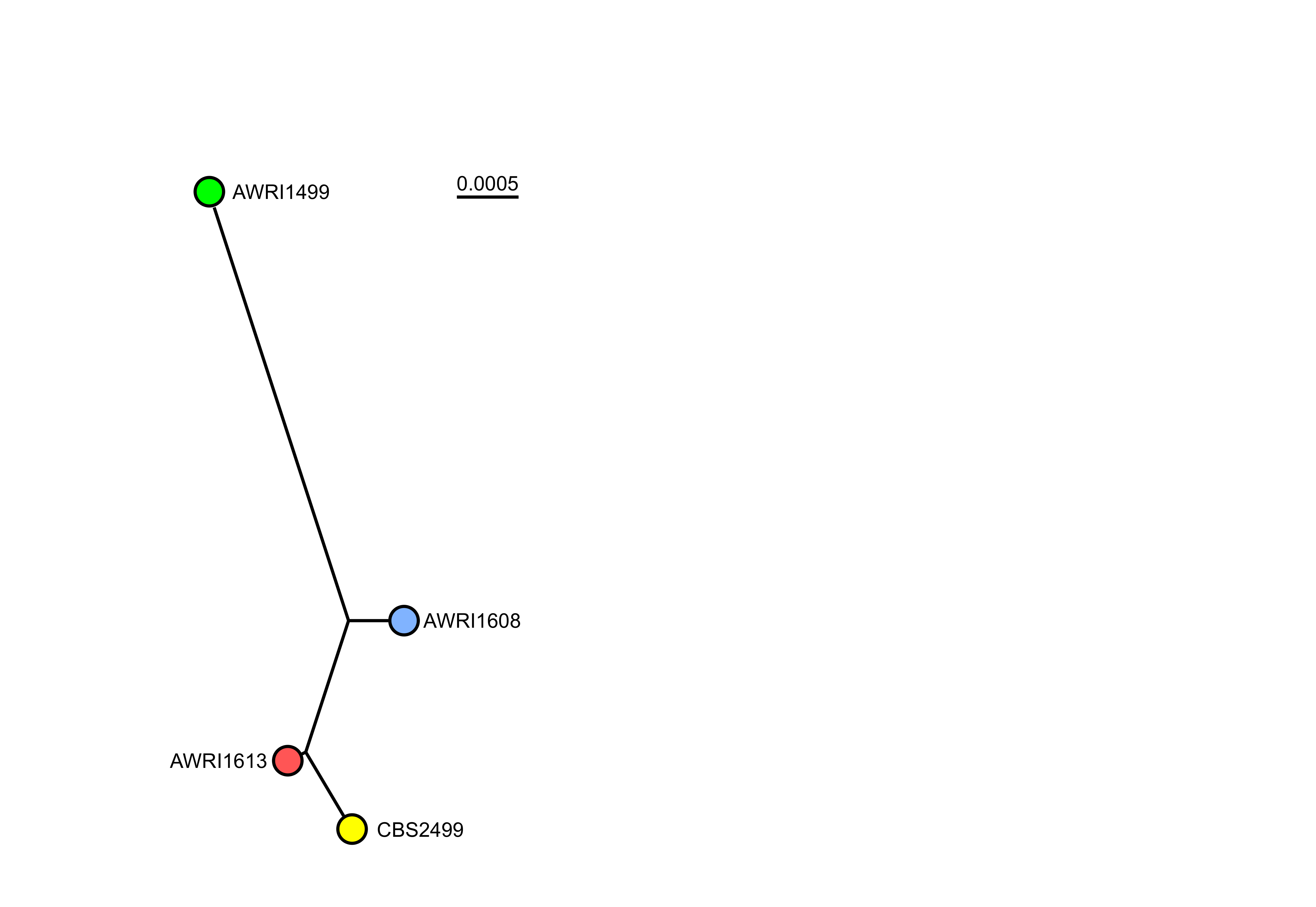

Supplement: Figure S2 — Whole genome phylogenies of D. bruxellensis. Whole-genome alignments were produced for each strain by converting nucleotides within the AWRI1499 reference based upon the results of the read-mapping and SNP analysis. Any regions displaying nucleotide insertions or deletions or low read coverage (<10 reads) in at least one strain were then removed prior to further analysis. The maximum-likelihood phylogeny was then calculated for these alignments using PhyML. (TIF) [file pgen.1004161.s002.tif]

A.

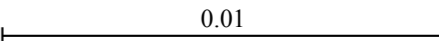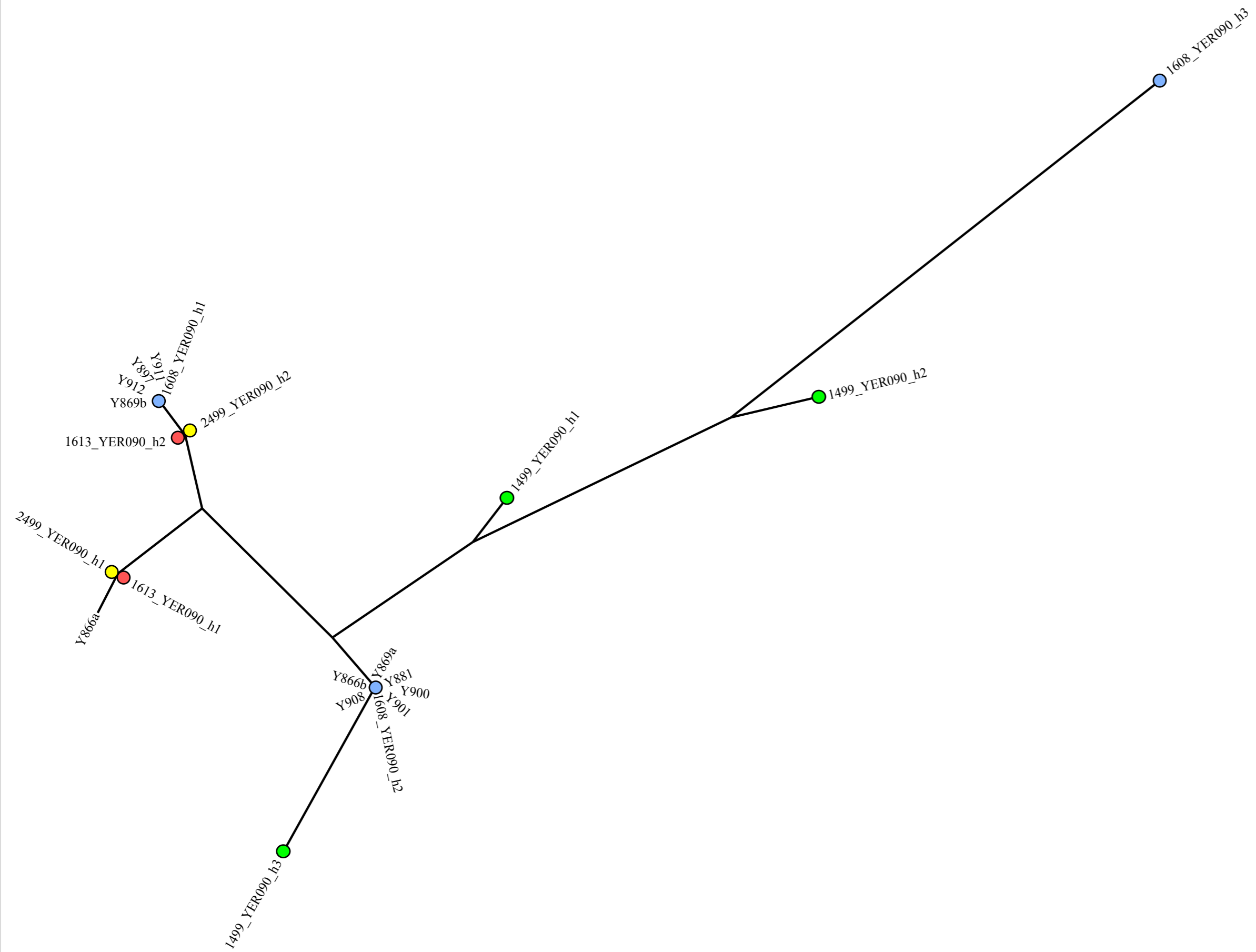

B.

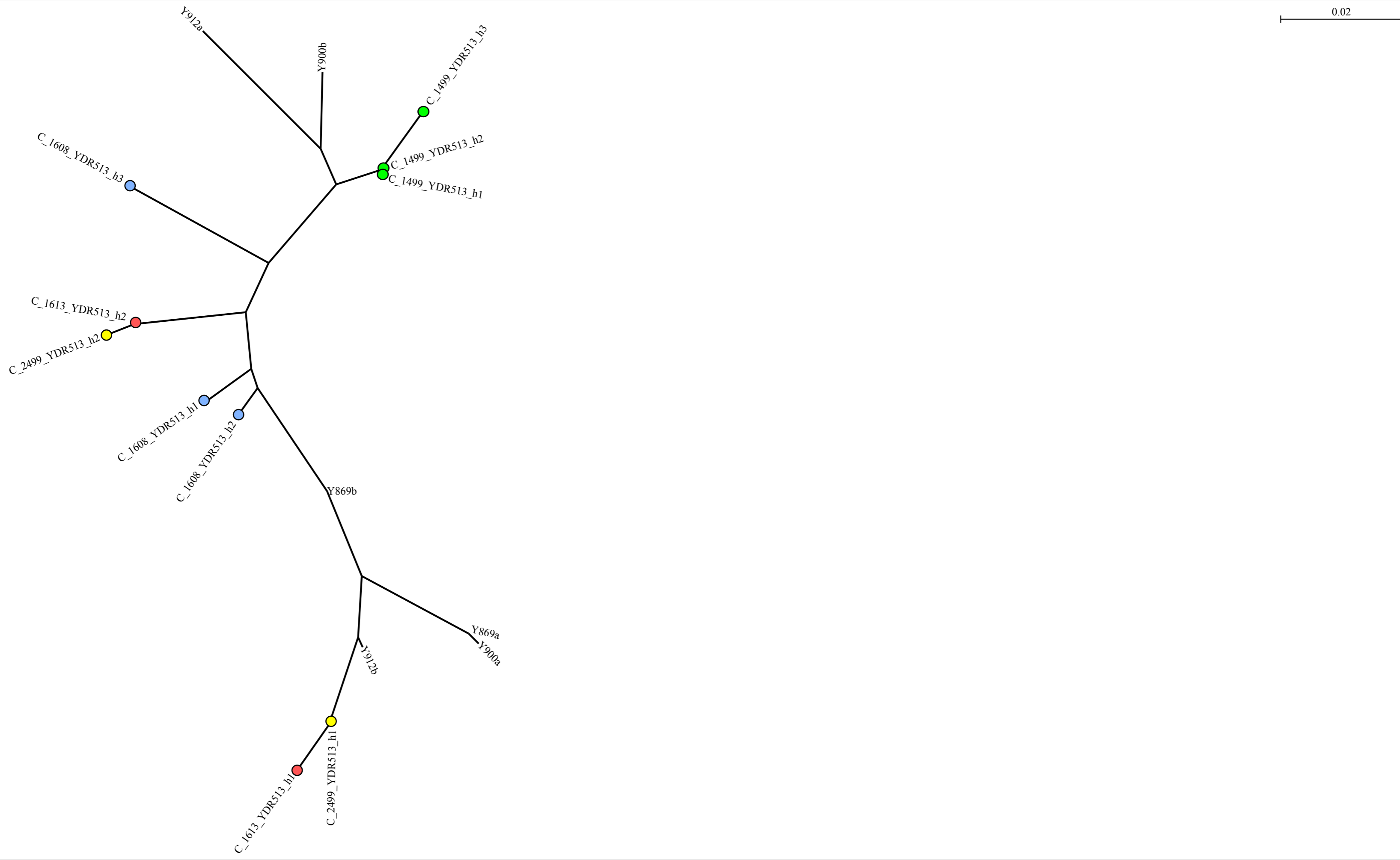

C.

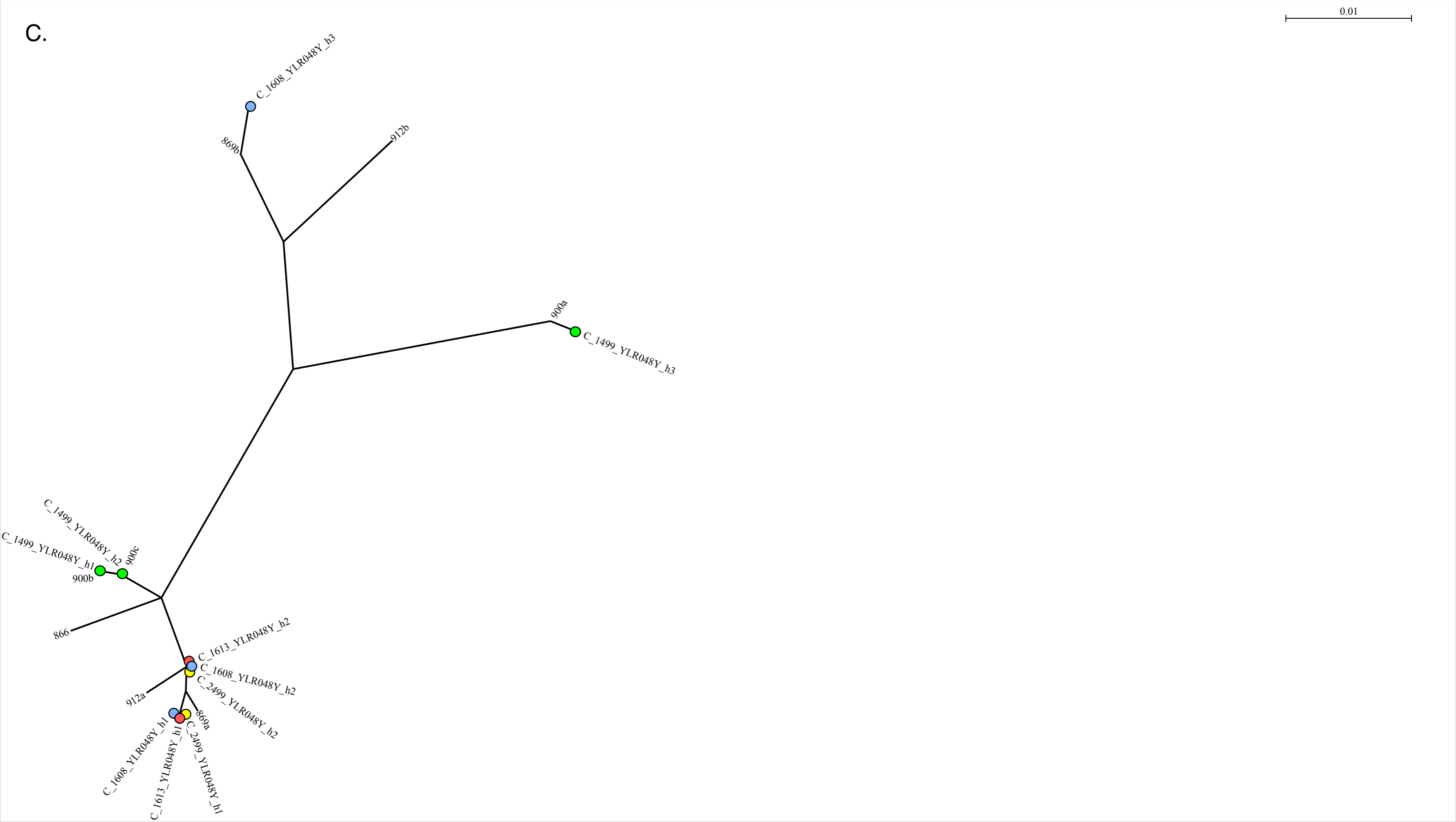

D.

0.01

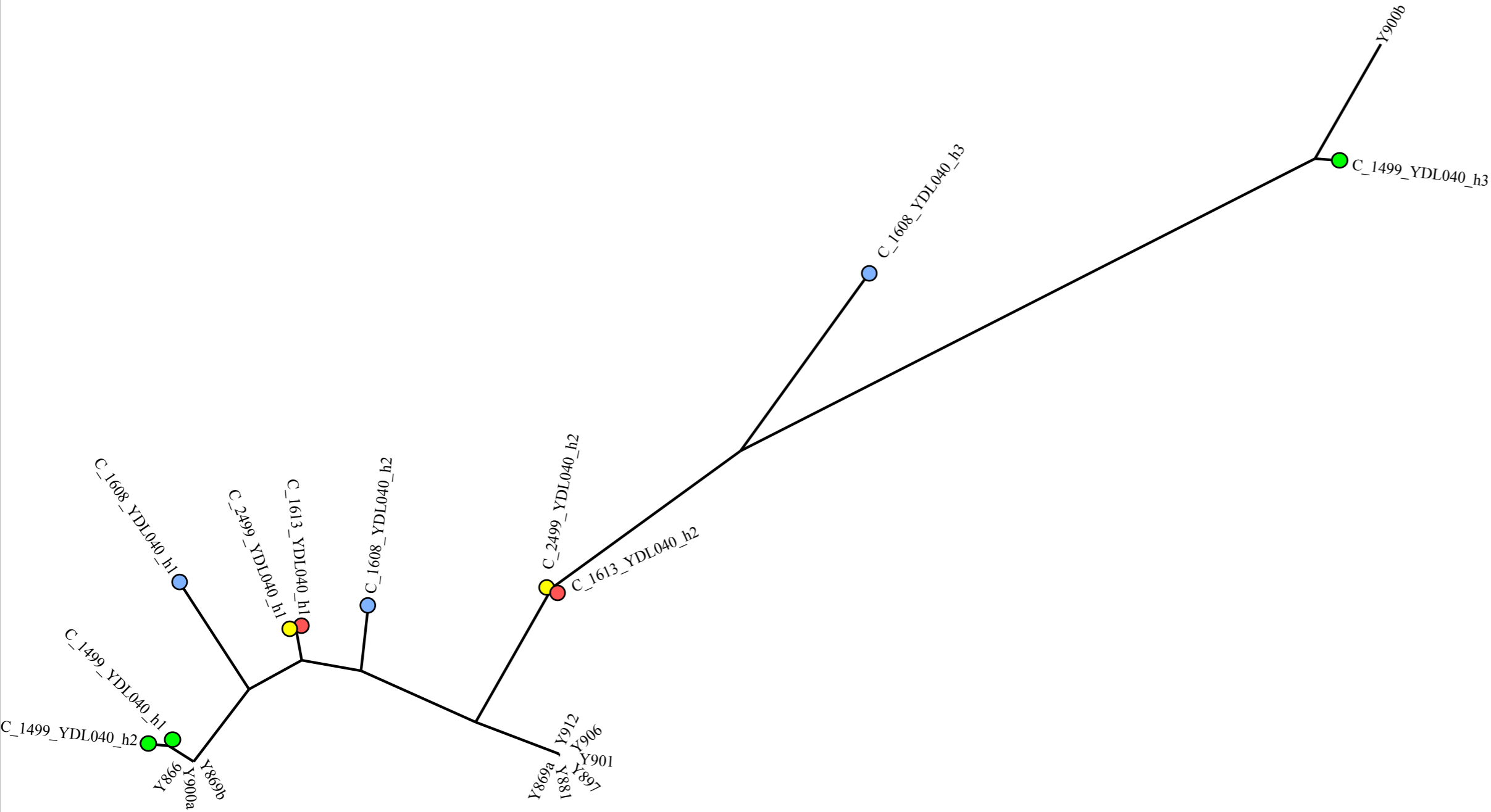

E.

0.005

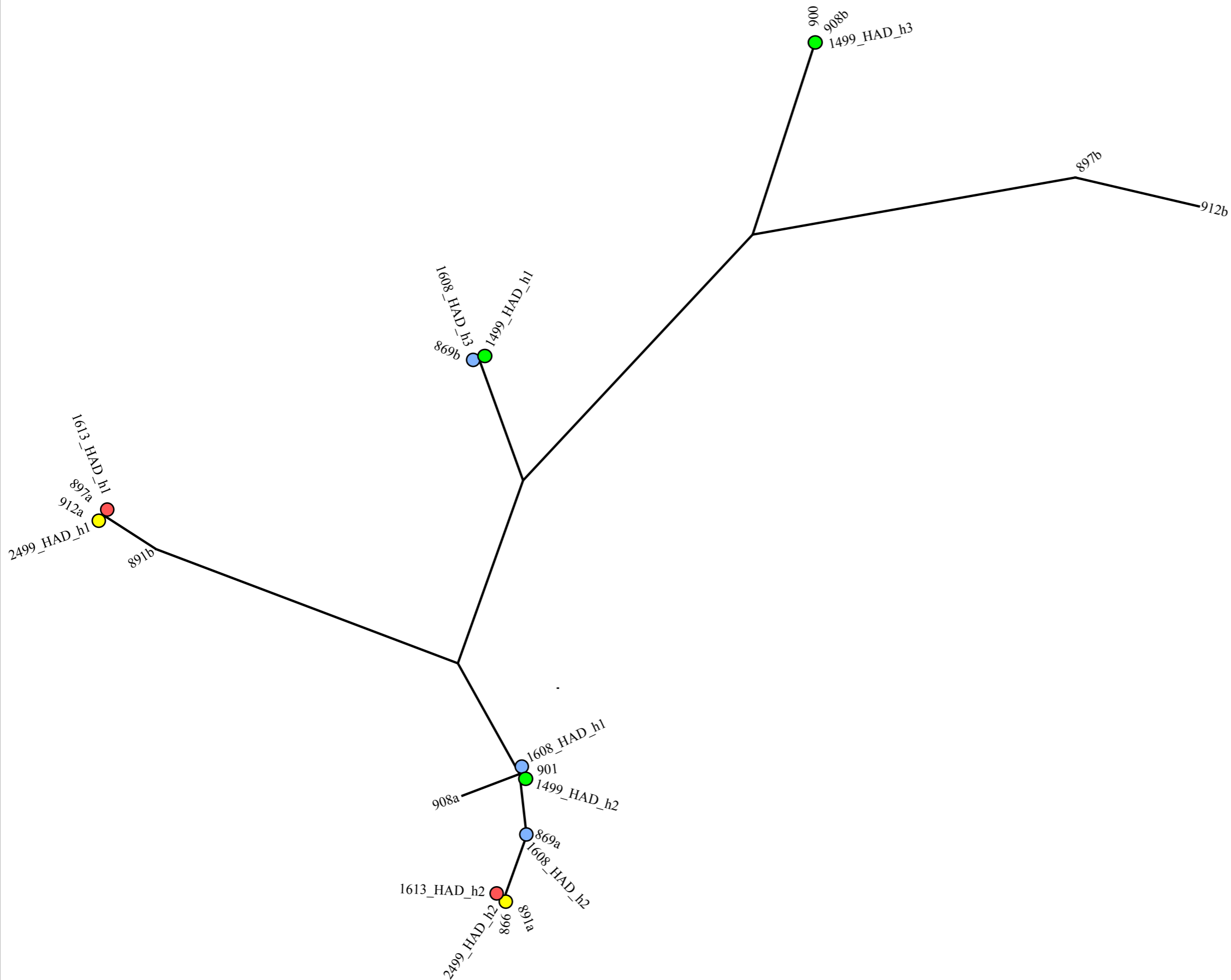

Supplement: Figure S3 — Haplotype analysis for additional D. bruxellensis isolates. Distinct haplotypes were assembled for genomic regions of AWRI1499, AWRI1608, AWRI1613 and CBS2499 that matched loci studied by Hellborg and Piškur [17] (data downloaded from NCBI for additional strains on 21 October 2013). Haplotype sequences (Dataset S3) for (A) DbYER090, (B) DbYDR513, (C) DbYLR048Y, (D) DbYDL040 and (E) DbHAD1 were subjected to maximum-likelihood phylogenetic analysis [15], [16]. Nodes are color-coded according to strain AWRI1499 (green), AWRI1608 (blue), AWRI1613 (red), CBS2499 (yellow). (PDF) [file pgen.1004161.s003.pdf]

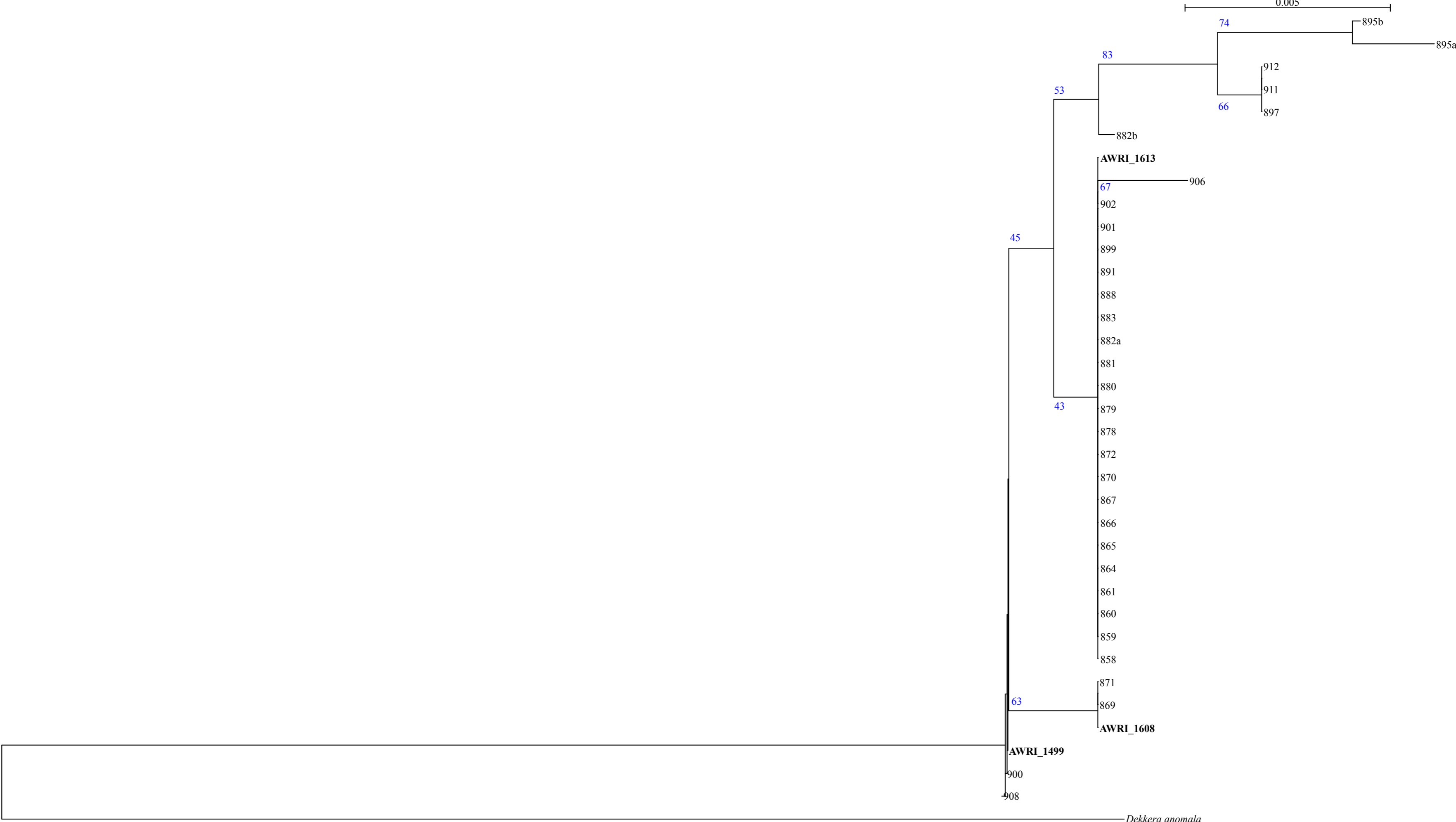

Supplement: Figure S4 — Phylogenic relationship of D. bruxellensis isolates. 26S rDNA (D1/D2 domain) sequences for AWRI1499, AWRI1608 and AWRI1613 [1] and 30 other D. bruxellensis isolates [17] were aligned and a neighbor-joining tree constructed after removal of all gapped bases [15], with D. anomala as the outgroup. Bootstrap support from 1000 randomisations indicated in blue. Isolates exhibiting more than one ribotype denoted by different letters. (PDF) [file pgen.1004161.s004.pdf]

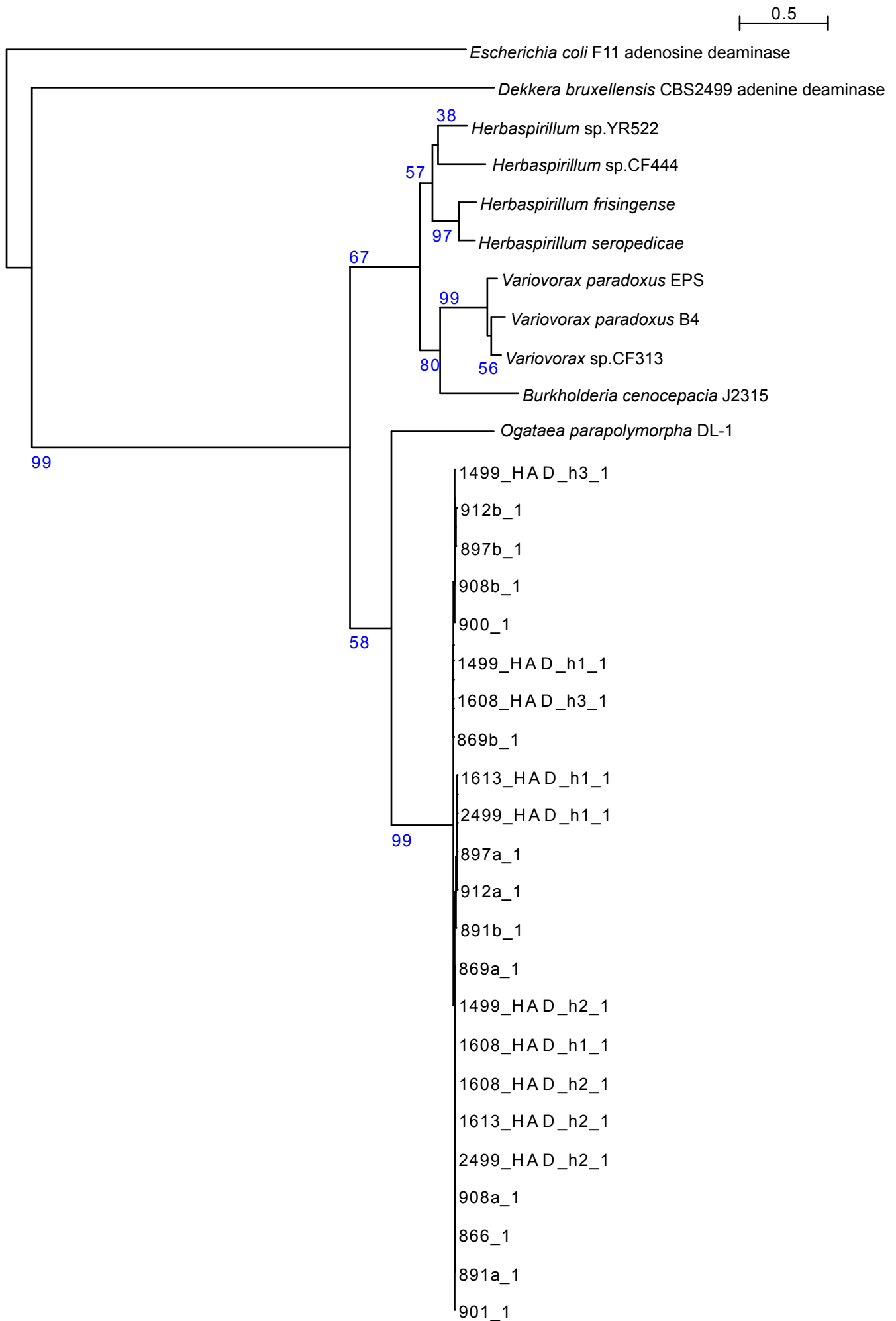

Supplement: Figure S5 — Phylogenic analysis of horizontally transferred adenyl deaminase. Maximum-likelihood phylogenies were produced for haplotype-resolved predicted protein sequences of DbHAD1 for multiple D. bruxellensis isolates [17], in addition to the best matches present in the Genbank non-redundant protein database (at 21 October 2013) and the canonical D. bruxellensis adenine deaminase (DbADE1) [10]. Escherichia coli adenosine deaminase was used as the outgroup, and bootstrap support from 1000 randomisations is indicated in blue. (PDF) [file pgen.1004161.s005.pdf]

0.2

AWRI1499\_1134

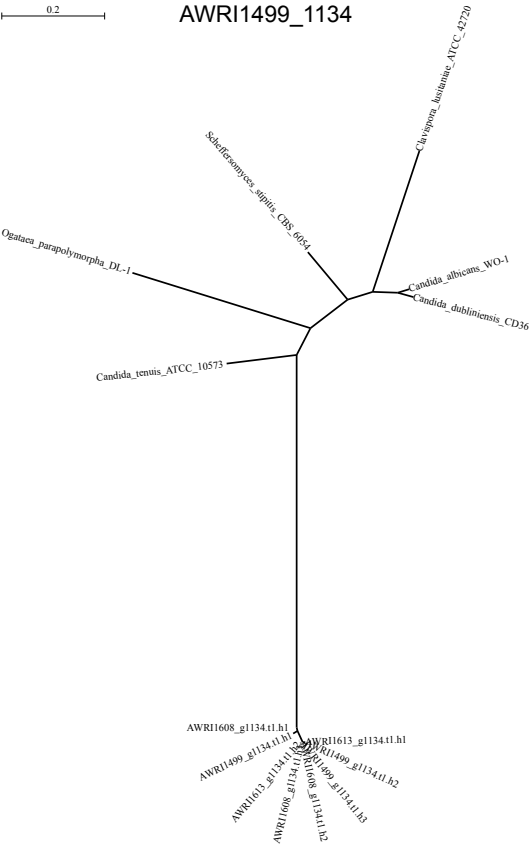

0.2

AWRI1499\_1822

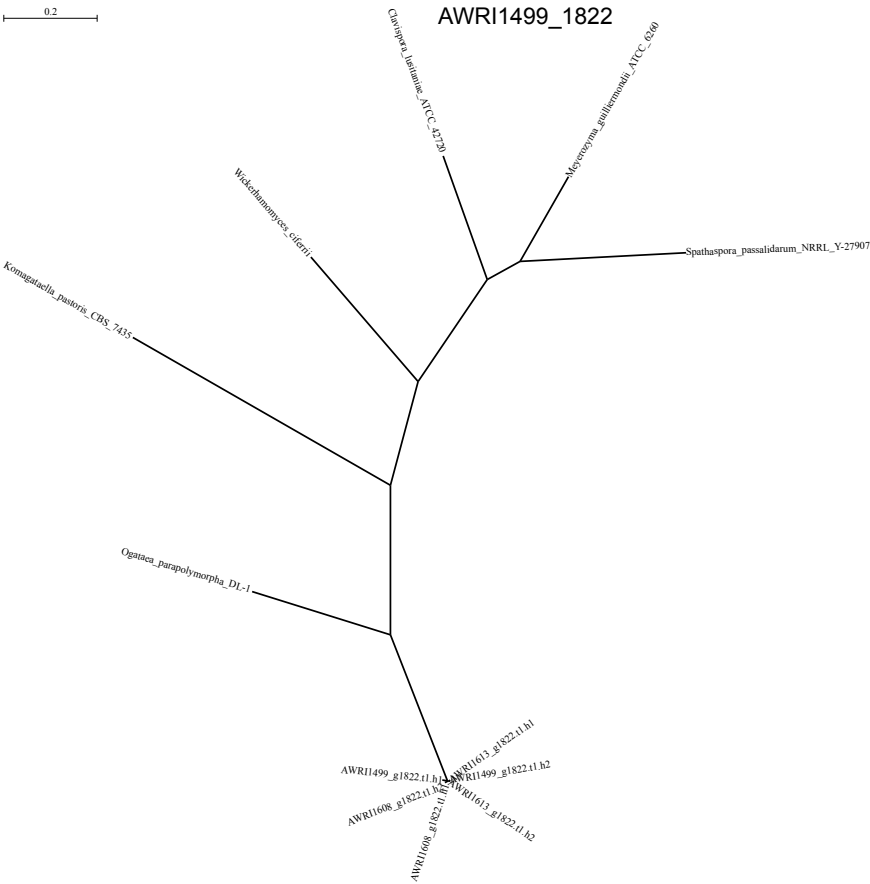

0.2

AWRI1499\_4264

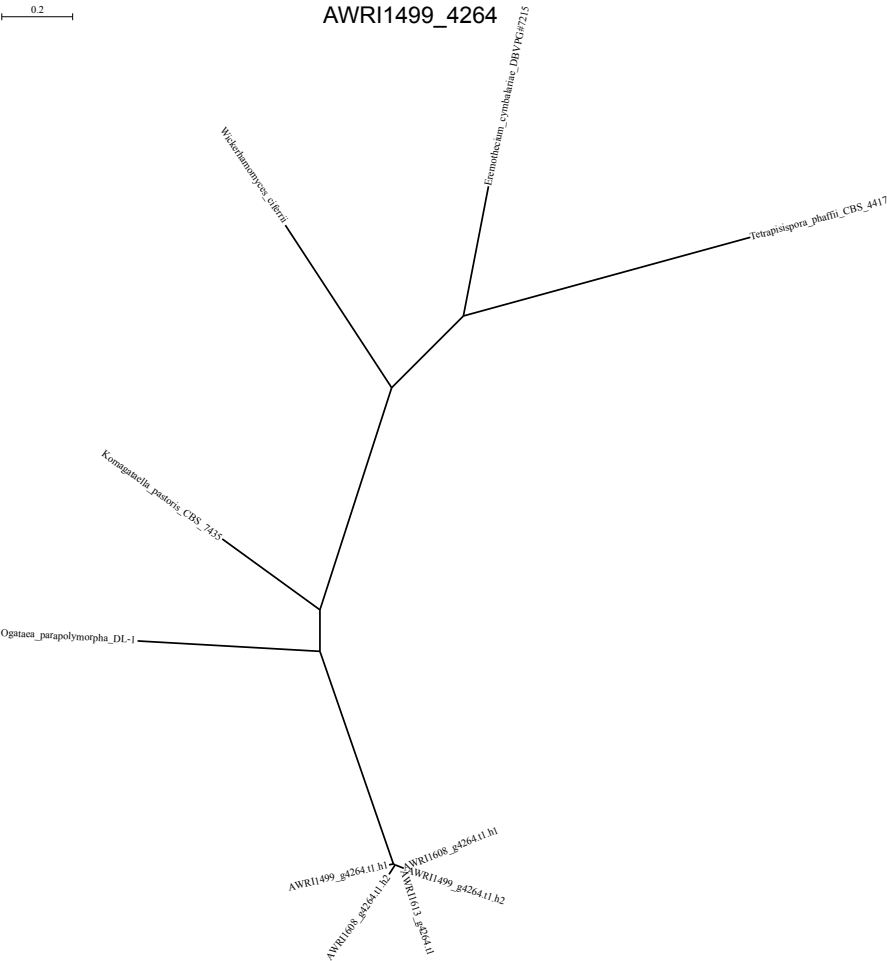

Supplement: Figure S6 — Broader phylogenic analysis of D. bruxellensis proteins. Maximum-likelihood phylogenies were produced for haplotype-resolved predicted protein sequences of three D. bruxellensis ORFs in addition to the best matches present in the Genbank non-redundant protein database. (PDF) [file pgen.1004161.s006.pdf]

**A**

PhyML ln(L)=-35189.5 1545 sites LG 4 rate classes

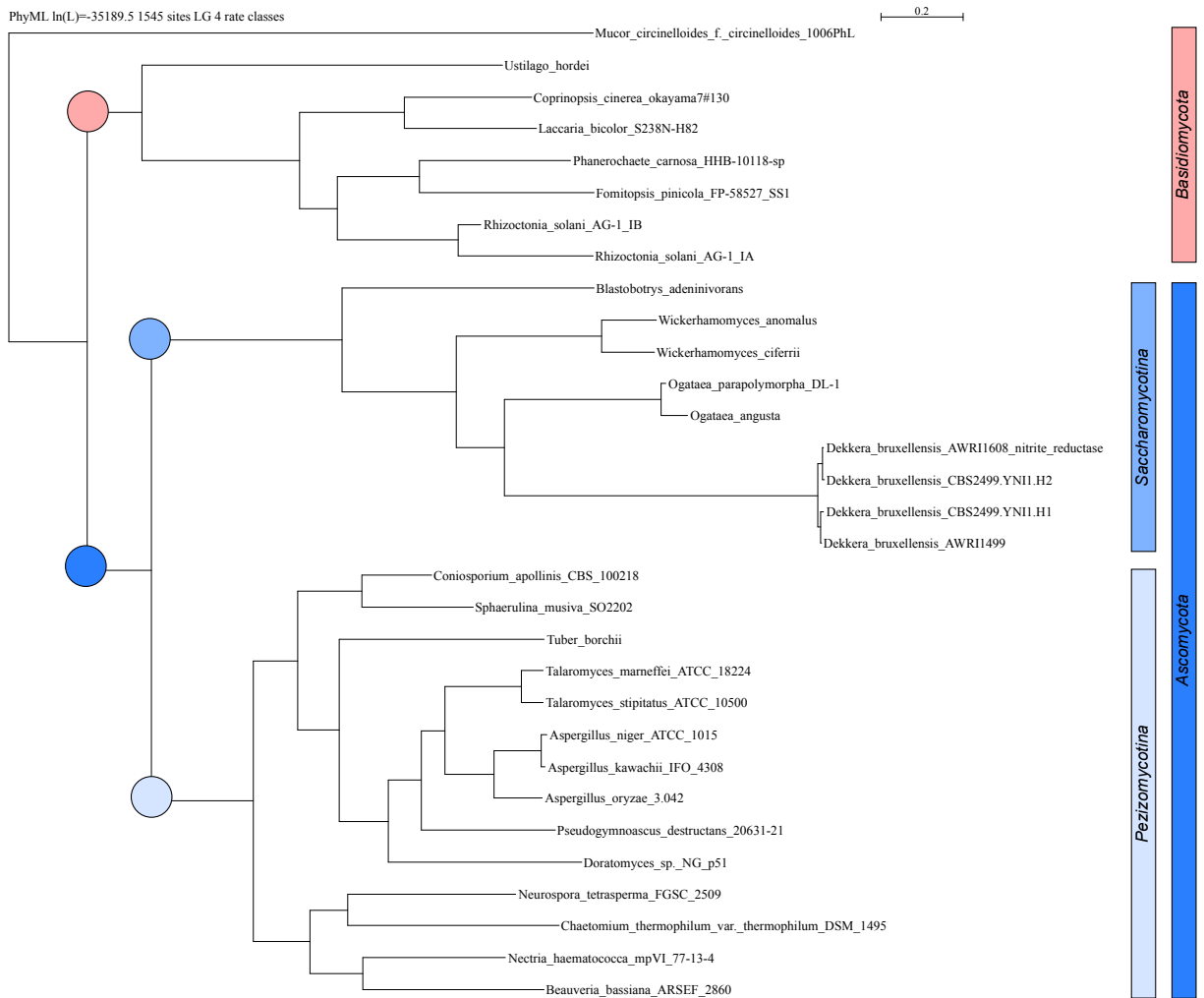**B**

PhyML ln(L)=-35845.6 1932 sites LG 4 rate classes

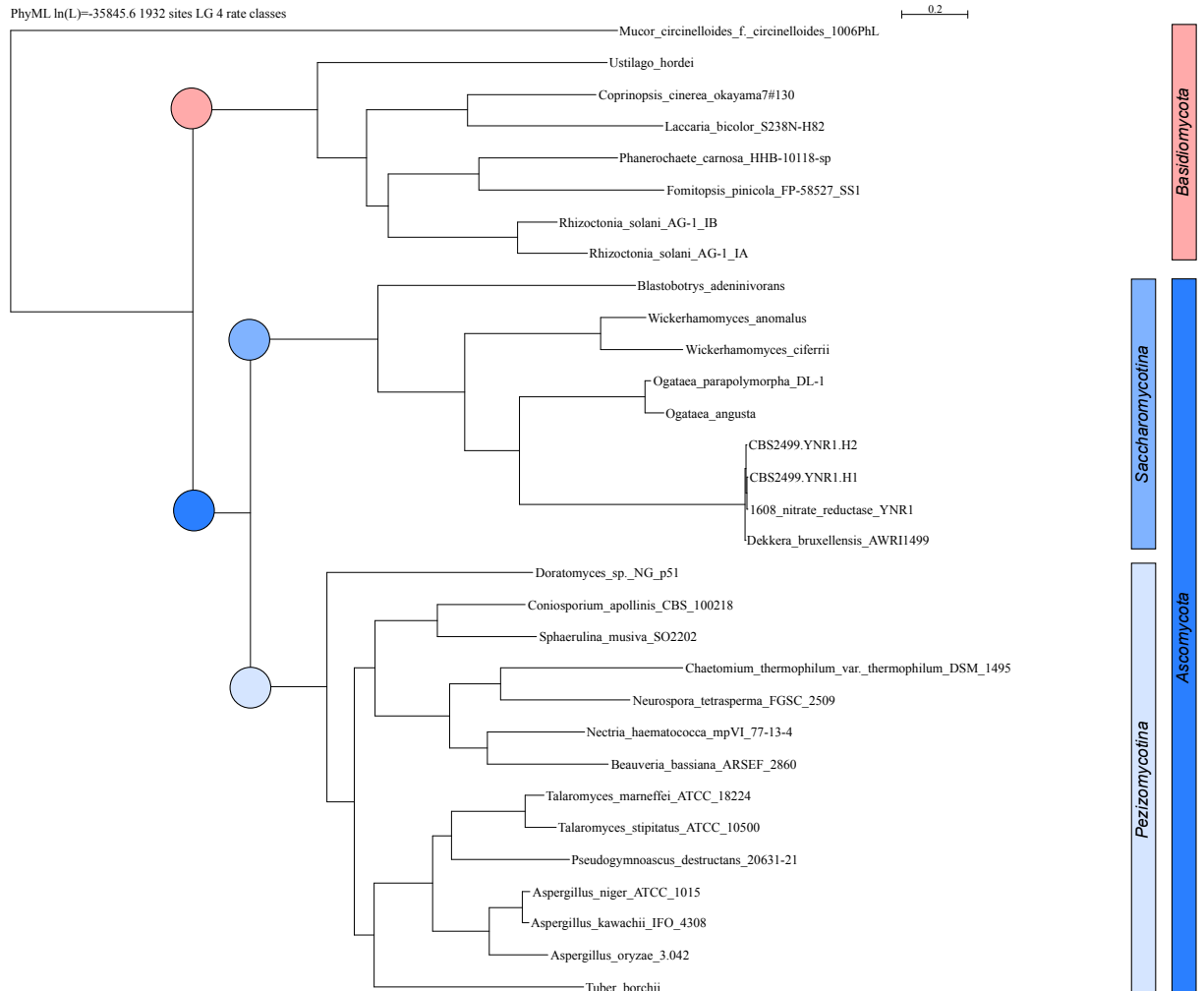

Supplement: Figure S7 — Phylogenic analysis of the nitrate assimilation cluster in D. bruxellensis. Maximum-likelihood phylogenies were prepared separately for both nitrite (A) and nitrate (B) reductases. All homologous protein sequences from the nr Genbank dataset for members of the Saccharomycotina subphylum are included, in addition to representative sequences from both Pezizomycotina and Basidiomycota. Sequences from Mucor circinelloides were included as an outgroup. (PDF) [file pgen.1004161.s007.pdf]

PhyML ln(L)=-2479.3 1602 sites GTR 4 rate classes

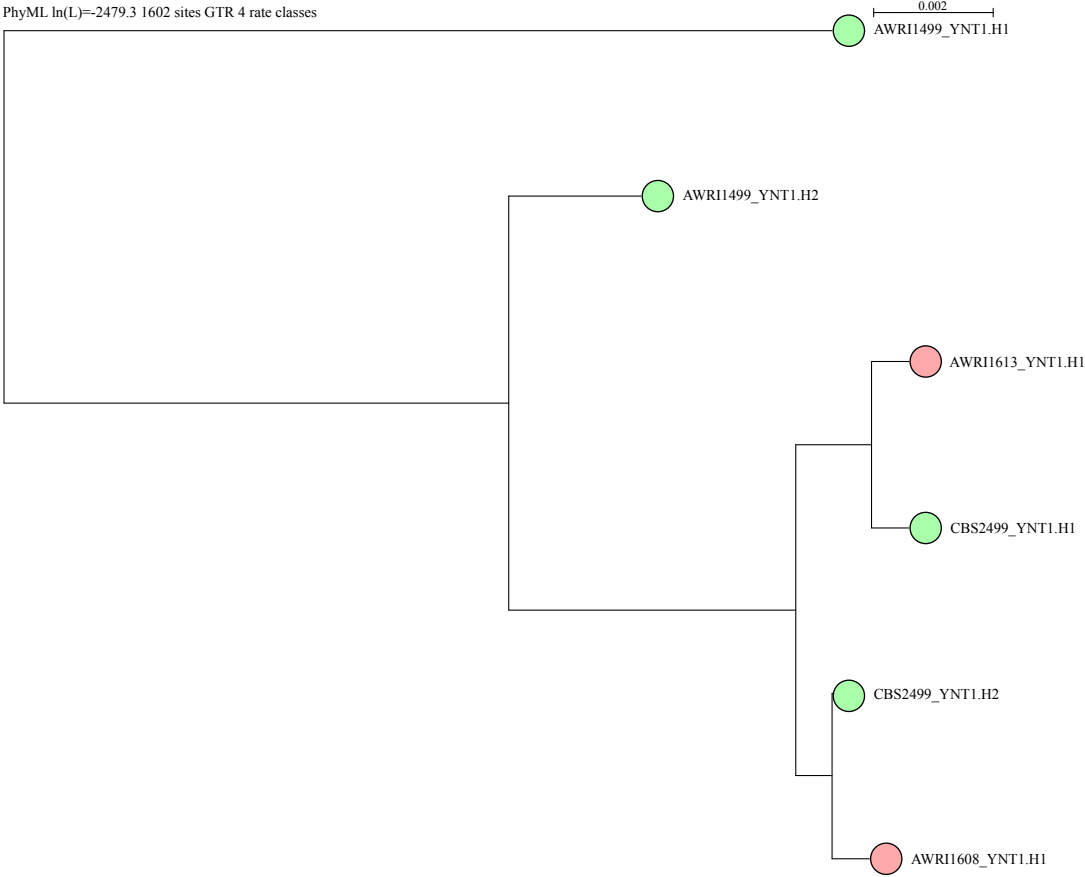

Supplement: Figure S8 — Phylogenic analysis of the predicted nitrate transporter of D. bruxellensis. A maximum-likelihood phylogeny was constructed from the nucleotide sequence of haplotype-resolved ORFs from AWRI1499, AWRI1608, AWRI1613 and CBS2499. Node colors represent the ability of the parent strain to utilize nitrate as a nitrogen source (green - growth; red - no growth). (PDF) [file pgen.1004161.s008.pdf]
