# Supplementary material for: Insights into the Dekkera bruxellensis Genomic Landscape: Comparative Genomics Reveals Variations in Ploidy and Nutrient Utilisation Potential amongst Wine Isolates
Source: PLoS Genet. 2014 Feb 13;10(2):e1004161. doi: 10.1371/journal.pgen.1004161 (PMC3923673; doi:10.1371/journal.pgen.1004161)
Supplement: Dataset S4 — Protein-based alignments of the nitrate reductase cluster. Clustal alignments were produced for the predicted nitrate reductase (YNR1), nitrite reductase (YNI1) and nitrate transporter (YNT1) proteins from AWRI1608 and CBS2499. (DOCX) [file pgen.1004161.s012.docx]

CLUSTAL O(1.1.0) multiple sequence alignment of AWRI1608 and CBS 2499 *YNR1*

AWRI1608.YNR1 MIMASSAKTDMITDIGVTKEEFENTYGNLADIFRLPFPVRRDKISIKPPNPKGTAEDDRV

CBS2499.YNR1.H1 --MASSAKTDMITDIGVTKEEFENTYGNLADIFRLPFPVRRDKISIKPPNPKGTAEDDRV

CBS2499.YNR1.H2 --MASSAKTDMITDIGVTKEEFENTYGNLADIFRLPFPVRRDKISIKPPNPKGTAEDDRV

**********************************************************

AWRI1608.YNR1 PRDKSLLRLTGVQPFNAEAPLDQLYDCGFLTKVGLHYVRNHGGVPEISEDIMNWEVEVGG

CBS2499.YNR1.H1 PRDKSLLRLTGVQPFNAEAPLDQLYDCGFLTKVGLHYVRNHGGVPEISEDIMNWEVEVGG

CBS2499.YNR1.H2 PRDKSLLRLTGVQPFNAEAPLDQLYDCGFLTKVGLHYVRNHGGVPKISEDIMNWEVEVGG

*********************************************:**************

AWRI1608.YNR1 MVEHPFKFKLKEIISDYDIYTVPVSLTCAGNRRKEQNMVKRGTGFNWGSAGTSTSLWTGC

CBS2499.YNR1.H1 MVEHPFKFKLKEIISDYDIYTVPVSLTCAGNRRKEQNMVKRGTGFNWGSAGTSTSLWTGC

CBS2499.YNR1.H2 MVEHPFKFKLKEIISDYDIYTVPVSLTCAGNRRKEQNMVKRGTGFNWGSAGTSTSLWTGC

************************************************************

AWRI1608.YNR1 MMRDIMLKAKPKNAARFVWMEGGDNPANGPYGTSVRLCWVMDPERSIMLAYKQNGSFLSP

CBS2499.YNR1.H1 MMRDIMLKAKPKNAARFVWMEGGDNPANGPYGTSVRLCWVMDPERSIMLAYKQNGSFLSP

CBS2499.YNR1.H2 MMRDIMLKAKPKNAARFVWMEGGDNPANGPYGTSVRLCWVMDPERSIMLAYKQNGSFLSP

************************************************************

AWRI1608.YNR1 DHGRPFRAIIPGVIGGRSVKWLKKIIISDVPSNNWYHYFDNRVLPTMVTPEEAKKNKQWW

CBS2499.YNR1.H1 DHGRPFRAIIPGVIGGRSVKWLKKIIISDVPSNNWYHYFDNRVLPTMVTPEEAKKNKQWW

CBS2499.YNR1.H2 DHGRPFRAIIPGVIGGRSVKWLKKIIISDVPSNNWYHYFDNRVLPTMVTPEEAKKNKQWW

************************************************************

AWRI1608.YNR1 KDERYALYDMAVQSAIWKPSNNETVAVDGEDDDNTVSVSGYASNGAGVRVGRVEVSLDKG

CBS2499.YNR1.H1 KDERYALYDMAVQSAIWKPSNNETVAVSGEDDDNTVSVSGYASNGAGVRVGRVEVSLDKG

CBS2499.YNR1.H2 KDERYALYDMAVQSAIWKPSNNETVTVDGEDDDNTVSVSGYASNGAGVRVGRVEVSLDKG

*************************:*.********************************

AWRI1608.YNR1 RTWKLADIDYPEDRYREAGHIKLFGGLVNVCDRMSCLCWCFWKISIPKKELLVAKDIVVR

CBS2499.YNR1.H1 RTWKLADIDYPEDRYREAGHIKLFGGLVNVCDRMSCLCWCFWKISIPKKELLVAKDIVVR

CBS2499.YNR1.H2 RTWKLADIDYPEDRYREAGHIRLFGGLVNVCDRMSCLCWCFWKISIPKKELFVAKDIVVR

*********************:*****************************:********

AWRI1608.YNR1 AMDERMCIQPRDMYWNVTSMMNNWWYRVAIRKDGPNVIRFEHPVLANKKGGWMDRVKSEG

CBS2499.YNR1.H1 AMDERMCIQPRDMYWNVTSMMNNWWYRVAIRKDGPNVIRFEHPVLANKKGGWMDRVKSEG

CBS2499.YNR1.H2 AMDERMCIQPRDMYWNVTSMMNNWWYRVAIRKDGPNVIRFEHPVLANKKGGWMDRVKSEG

************************************************************

AWRI1608.YNR1 GDFLDGNWGENDGAGFGRSDSTRRKPVVDTDLQLISNPEKMKVIITKEEFDKHGKNISDS

CBS2499.YNR1.H1 GDFLDGNWGENDGAGFGRSDSTRRKPVVDTDLQLISNPEKMKVIITKEEFDKHGKNISDS

CBS2499.YNR1.H2 GDFLDGNWGENDGAGFGRSDSTRRKPVVDTDLQLISNPEKMKVIITKEEFDKHGKNISDS

************************************************************

AWRI1608.YNR1 NLNPWFLVKGLVFDGSSFLDDHPGGAEAILGVTGEDATDQFMSIHSDDAKRMLATMQVGV

CBS2499.YNR1.H1 NLNPWFLVKGLVFDGSSFLDDHPGGAEAILGVTGEDATDQFMSIHSDDAKRMLATMQVGV

CBS2499.YNR1.H2 NLNPWFLVKGLVFDGSSFLDDHPGGAEAILGVTGEDATDQFMSIHSDDAKRMLATMQVGV

************************************************************

AWRI1608.YNR1 LEGFSQDTNDNEENGDLTPGATFLRPKFWKKVELISREDVSEDSAIFTFKLETPEQKFGL

CBS2499.YNR1.H1 LEGFSQDTNDNEENGDLTPGATFLRPKFWKKVELISREDVSEXSAIFTFKLETPEQKFGL

CBS2499.YNR1.H2 LEGFSQDTNDNEENGDLTPGATFLRPKFWKKVELISREDVSEXSAIFTFKLETPEQKFGL

****************************************** *****************

AWRI1608.YNR1 PCRKARVCENE

CBS2499.YNR1.H1 PCRKARVCENE

CBS2499.YNR1.H2 PCRKARVCENE

***********

CLUSTAL O(1.1.0) multiple sequence alignment of AWRI1608 and CBS 2499 *YNI1*

AWRI1608.YNI1 MSDLKPGIPPNPEDIHPPSQKLSIVVVGLGMVGFSFLQKILDLDQEQNKYIITIIGEERY

CBS2499.YNI1.H1 MSDLKPGIPPNPEDIHPPSQKLSIVVVGLGMVGFSFLQKILDLDQEQNKYIITVIGEERY

CBS2499.YNI1.H2 MSDLKPGIPPNPEDIHPPSQKLSIVVVGLGMVGFSFLQKILDLDQEQNKYIITIIGEERY

*****************************************************:******

AWRI1608.YNI1 LAYNRVKLTDYFLHRNVDELLLSDKEFYEKHDSKKWGYFVDDAVVSINRKSKTVTTASDR

CBS2499.YNI1.H1 LAYNRVKLTDYFLHRNVNELLLSDKEFYEKRDSKKWGYFVDDAVVSINRKSKTVTTASDR

CBS2499.YNI1.H2 LAYNRVKLTDYFLHRNVDELLLSDKEFYEKHDSKKWGYFVDDAVVSINRKSKTVTTASDR

*****************:************:*****************************

AWRI1608.YNI1 IVKYDKLVLATGSDARAPGDVHDKIKAKNMGCYVYRTIDDLNQLIHYTEQNINDDKAHAI

CBS2499.YNI1.H1 VVKYDKLVLATGSDARAPGDVHDKIKAKNMGCYVYRTIDDLNQLIHYTEQNFNDDKAHAI

CBS2499.YNI1.H2 IVKYDKLVLATGSDARAPGDVHDKIKAKNMGCYVYRTIDDLNQLIHYTEQNFNDDKAHAI

:**************************************************:********

AWRI1608.YNI1 IVGGGLLALEAAKAVMDLGKFDSIKIVQLDGWLMGRQLDKRGGKLLEKKISKMGIKVECG

CBS2499.YNI1.H1 IVGGGLLALEAAKAVMDLGKFDSIKIVQLDGWLMGRQLDKRGGKLLEKKISKMGIKVECG

CBS2499.YNI1.H2 IVGGGLLALEAAKAVMDLGKFDSIKIVQLDGWLMGRQLDKRGGKLLEKKISKMGIKVECG

************************************************************

AWRI1608.YNI1 LTTKSLIFNDDGHLSAVEYSNGTKERCDLICFAIGIVPRDDLARSCGLDVGPRGGVIVDN

CBS2499.YNI1.H1 LTTKSLIFDDDGHLSAVEYSNGTKERCDLICFAIGIVPRDNLARSCGLDVGSRGGVMVDN

CBS2499.YNI1.H2 LTTKSLIFNDDGHLSAVEYSNGTKERCDLICFAIGIVPRDDLARSCGLDVGPRGGVIVDN

********:*******************************:********** ****:***

AWRI1608.YNI1 YMQTSDPDIYAIGECAAWKGRTYGLIAPGYAMADVLSFNLTQGEMHSLKAFAEPDTNTRL

CBS2499.YNI1.H1 YMQTSDPDIYAIGECAAWKGRTYGLIAPGYAMADVLSFNLTQGEMHSLKAFFEPDTNTRL

CBS2499.YNI1.H2 YMQTSDPDIYAIGECAAWKGRTYGLIAPGYAMADVLSFNLTQGEMHSLKAFCEPDTNTRL

*************************************************** ********

AWRI1608.YNI1 KLLGVDVAAFGDYFADTEGPKWLPKDCRKCGKGARALIYDNPIDEVYQKLIFTEDGRYLL

CBS2499.YNI1.H1 KLLGVDVAAFGDYFADTEGPKWLPKDCHKCGKGARALIYDNPIDEVYQKLIFTEDGRYLL

CBS2499.YNI1.H2 KLLGVDVAAFGDYFADTEGPKWLPKDCRKCGKGARALIYDNPIDEVYQKLIFTEDGRYLL

***************************:********************************

AWRI1608.YNI1 GGILVGDNSKYPLLSALTKKRKPLTQEPGELIIGKQSGDDSGDGVDALPDEAQICSCNNI

CBS2499.YNI1.H1 GGILVGDNSKYPLLSALTKKRKPLAQEPGELIIGKQSGDDSGNGVDALPDEAQICSCNNI

CBS2499.YNI1.H2 GGILVGDNSKYPLLSALTKKRKPLTQEPGELIIGKQSGDDSGDGVDALPDEAQICSCNNI

************************:*****************:*****************

AWRI1608.YNI1 SKGQIVQAIKNGSTTLDLIKKNTKAGTACGGCVPTLKLILESELKKMGKKVSTDLCVHFK

CBS2499.YNI1.H1 SKGQIVQAIKNGSTTLELIKKNTKAGTACGGCVPTLKLILESELKKMGKEVSTDLCVHFK

CBS2499.YNI1.H2 SKGQIVQAIKNGSTTLDLIKKNTKAGTACGGCVPTLKLILESELKKMGKKVSTDLCVHFK

****************:********************************:**********

AWRI1608.YNI1 YSRSDLFSIIMVKRYKTFGEVMENLGTTPDSSGCEICKPTIGSILSTLYGRHLMEKEFQG

CBS2499.YNI1.H1 YSRSDLFSIIMVKRYKTFGEVMENLGTTPDSSGCEICKPTIGSILSTLYGRHLMEKEFQG

CBS2499.YNI1.H2 YSRSDLFSIIMVKRYKTFGEVMENLGTTPDSSGCEICKPTIGSILSTLYGRHLMEKEFQG

************************************************************

AWRI1608.YNI1 LQETNDRYLGNLQRNGTYSVVPRMSAGEITPEKLISIGKVAKKYDLYTKITGGQRVDLFG

CBS2499.YNI1.H1 LQETNDRYLGNLQRNGTYSVVPRMSAGEITPEKLISIGKVAKKYDLYTKITGGQRVDLFG

CBS2499.YNI1.H2 LQETNDRYLGNLQRNGTYSVVPRMSAGEITPEKLISIGKVAKKYDLYTKITGGQRVDLFG

************************************************************

AWRI1608.YNI1 VKKQDLLKVWEDLHKAGFESGQAYGKTLRNVKSCVGSTWCRYGIGDSVGLAIRLEQRYRG

CBS2499.YNI1.H1 VKKQDLLKVWEDLHKAGFESGQAYGKTLRNVKSCVGSTWCRYGIGDSVGLAIRLEQRYRG

CBS2499.YNI1.H2 VKKQDLLKVWEDLHKAGFESGQAYGKTLRNVKSCVGSTWCRYGIGDSVGLAIRLEQRYRG

************************************************************

AWRI1608.YNI1 IRSPHKIKGGVSGCVRDCAEYHSKDFGLCAVQGGFDIYVGGNGGMKPAAAQLLASKVKPD

CBS2499.YNI1.H1 IRSPHKIKGGVSGCVRDCAEYHSKDFGLCAVQGGFDIYVGGNGGMKPAAAQLLASKVKPD

CBS2499.YNI1.H2 IRSPHKIKGGVSGCVRDCAEYHSKDFGLCAVQGGFDIYVGGNGGMKPAAAQLLASKVKPD

************************************************************

AWRI1608.YNI1 MVIRILDRYLMFYLRTADRLQRTARWLEKLDGGIDYLKSVIIQDKLGIAEELEKQMQDIV

CBS2499.YNI1.H1 MVIRILDRYLMFYLRTADRLQRTARWLEKLEGGIDYLKSVIIQDKLGIAEELEKQMQDIV

CBS2499.YNI1.H2 MVIRILDRYLMFYLRTADRLQRTARWLEKLEGGIDYLKSVIIQDKLGIAEELEKQMQDIV

******************************:*****************************

AWRI1608.YNI1 SHYFDEWGRTLKEKDSEAPLFKQFANTDENQESVEMVYERGQRRPALWADEPAKMRFNEI

CBS2499.YNI1.H1 SHYFDEWGRTLKEKDSEAPLFKQFANTDENQESVEMVYERGQRRPALWADEPAKMRFNEI

CBS2499.YNI1.H2 SHYFDEWGRTLKEKDSEAPLFKQFANTDENQESVEMVYERGQRRPALWADEPAKMRFNEI

************************************************************

AWRI1608.YNI1 KWSSSHWKKGIEEQ

CBS2499.YNI1.H1 KWSSSHWKKGSEEQ

CBS2499.YNI1.H2 KWSSSHWKKGIEEQ

********** ***

CLUSTAL O(1.1.0) multiple sequence alignment of AWRI1608 and CBS 2499 *YNT1*

CBS.YNT1 MWRISSLWKXAVVNQRNKKSATIPILNVFDIYGRNFLFAWLGFLVCFLSWFGVPPLMTKM

AWRI1608.YNT1 MWRISSLWKPAVVNQRNKKSATIPILNVFDIYGRNFLFAWLGFLVCFLSWFGVPPLMTKM

********* **************************************************

CBS.YNT1 IKRDLKLTAVDIANNNICGLSATLLGRFVMGPVCDRYGPRWGMISILLIGAIPTAFMPLV

AWRI1608.YNT1 IKRDLKLTAVDIANNNICGLSATLLGRFVMGPVCDRYGPRWGMISILLIGAIPTAFMPLV

************************************************************

CBS.YNT1 NNVSGLHAIRFFISILGSSFVCCAQYVNAFFDNNIIGTANAVAAGWGNSGAGIAFFVMPA

AWRI1608.YNT1 NNVSGLHAIRFFISILGSSFVCCAQYVNAFFDNNIIGTANAVAAGWGNSGAGIAFFVMPA

************************************************************

CBS.YNT1 IASALLNNDGYSLHKAWSLSFVIGPFLILLFVAFLLLFFGQDCPMGKWSRRSDILGVNQN

AWRI1608.YNT1 IASALLNNDGYSLHKAWSLSFVIGPFLILLFVAFLLLFFGQDCPMGKWSRRSDILGVNQN

************************************************************

CBS.YNT1 NXLIKTVSLSKHGKVLSITASAVGVTGVDDPAAANKILVNKDSNDEKKYLNDEAQDSEEN

AWRI1608.YNT1 NTLIKTVSLSKHGKVLSITASAVGVTGVDDPAAANKILVNKDSNDEKKYLNDEAQDSEEN

* **********************************************************

CBS.YNT1 DVDIEDLISKDEIIRDPTFWDVAKISFAPRTLLCALPYLTTFGTELAVESILSALYEQHE

AWRI1608.YNT1 DVDIEDLISKDEIIRDPTFWDVAKISFAPRTLLCALPYLTTFGTELAVESILSALYEQHE

************************************************************

CBS.YNT1 KLWPMKKAGDWASMMGLLNVVTRPLGGFISDILYHHFKSTKAKKFWMLFCGVVQGIFLIW

AWRI1608.YNT1 KLWPMKKAGDWASMMGLLNVVTRPLGGFISDILYHHFKSTKAKKFWMLFCGVVQGIFLIW

************************************************************

CBS.YNT1 IGFRPHLSIAGLITALSFMAXFMEMANGANFAVVPFINKAHTGLIXGSTGAFGNAGGIFF

AWRI1608.YNT1 IGFRPHLSIAGLITALSFMALFMEMANGANFAVVPFINKAHTGLISGSTGAFGNAGGIFF

******************** ************************ **************

CBS.YNT1 SLVFRFTIVNGKNNYFRGFWIIGICSIVVNLAVCLIPIREERPKKIRTEMSCA

AWRI1608.YNT1 SLVFRFTIVNGKNNYFRGFWIIGICSIVVNLAVCLIPIREERPKKIRTEMSCA

*****************************************************
